# Interplay Between the Phenotype and Genotype, and Efflux Pumps in Drug-Resistant Strains of *Riemerella anatipestifer*

**DOI:** 10.3389/fmicb.2018.02136

**Published:** 2018-10-01

**Authors:** Qiwei Chen, Xiaowei Gong, Fuying Zheng, Guo Ji, Shengdou Li, Laszlo Stipkovits, Susan Szathmary, Yongsheng Liu

**Affiliations:** ^1^State Key Laboratory of Veterinary Etiological Biology, Lanzhou Veterinary Research Institute, Chinese Academy of Agricultural Sciences, Lanzhou, China; ^2^RT-Europe Research Center, Mosonmagyaróvár, Hungary

**Keywords:** *Riemerella anatipestifer*, efflux pump, efflux pump inhibitors, synergism, drug resistance

## Abstract

The number of multidrug-resistant strains of *Riemerella anatipestifer* continues to increase, and new strategies for the treatment of associated infections are necessary. Recently, numerous studies have shown that efflux pumps (EPs) play key roles in universal bacterial mechanisms that contribute to antibiotic resistance. In addition, studies have shown that the effects of antibiotics that are subjected to efflux can be reinforced by their combined use with efflux pump inhibitors (EPIs). Unfortunately, the role of the efflux system in *R. anatipestifer* remains barely understood. In this study, we evaluated the role of EPs and resistance genes in the resistance generated by clinical strains of *R. anatipestifer* to antibiotics. A set of 10 *R. anatipestifer* strains were characterized by drug resistance, associated resistance genes, and antibiotic profiles in the presence and absence of EPIs. Efflux activity was studied on a real time basis through a fluorometric method. Quantification of the levels of mRNA transcription of efflux pump genes (EPGs) was determined by RT-qPCR. Several approaches (detection of resistance genes, drug susceptibility testing, and growth kinetics analysis) were used to assess the correlation between the effect of the EPIs and the resistance levels. Analysis of the *R. anatipestifer* growth inhibition tests showed that the antibiotic activity was enhanced by the synergy of EPIs. Among the various resistance genes that confer antibiotic resistance, different minimum inhibitory concentrations (MICs) were observed. The different levels of resistance were reduced by EPIs. Real time fluorometry showed that all the *R. anatipestifer* strains presented inherent efflux activity, conferring varying levels of inhibition in the presence of EPIs. Moreover, 15 EPGs were overexpressed in the presence of antibiotics. The addition of EPIs to antibiotics led to downregulation in the expression of some EPGs and a simultaneous increase in drug resistance and sensitivity. These results demonstrated the contribution of these EPs in the resistant phenotype of the clinical strains of *R. anatipestifer* that are under investigation, independently of the resistant genotype of the respective strains. Intrinsic efflux activity was possibly linked to the evolution of resistance in multidrug-resistant isolates of *R. anatipestifer*. Furthermore, the inhibition of EPs by EPIs could enhance the clinical effects of antibiotics.

## Introduction

Duck serositis, caused by *Riemerella anatipestifer*, is an important communicable disease (Zheng et al., 2012; Guo et al., 2017). *R. anatipestifer* is a gram-negative, rod-shaped, non-spore-forming, non-motile bacterium that can primarily infect ducks that are l−5 weeks of age, *via* the respiratory or digestive tracts (Chikuba et al., 2016). Infection with this pathogen leads to great economic losses owing to the significant weight loss observed in ducklings and high mortality rates of up to 75% (Wang Q. et al., 2017). Thus far, at least 21 different serotypes of *R. anatipestifer* have been identified, and cross-immunoprotection among different serotypes has been barely reported (Zhai et al., 2013; Gyuris et al., 2017). All these factors complicate the prevention of this disease.

The molecular mechanisms associated with this bacterial infection remain largely unknown, and its treatment depends mainly on chemotherapy. However, the chronic use of antimicrobials has increasingly aggravated the resistance in *R. anatipestifer* isolates (Chang et al., 2003; Zhong et al., 2009). In previous studies (Chang et al., 2003; Chen et al., 2010, 2012; Xing et al., 2015), various antibiotic susceptibility tests of the field isolates of *R. anatipestifer* are reported. Nevertheless, studies on the molecular mechanisms of *R. anatipestifer* isolates that are resistant to antibiotics are limited. The resistance mechanisms associated with drug efflux pumps (EPs) have not been reported (Li, X. Z. et al., 2016).

Since the early 1990s, when the bacterial EPs were investigated for their significant contribution to multidrug resistance (MDR), a large number of studies have focused on efflux-mediated antimicrobial resistance and efflux determinants (Li et al., 2015). In bacteria, resistance can take many forms. Bacterial drug EPs constitute a major mechanism of both natural and acquired resistance to a diverse range of clinically used antibiotics and biocides. Based on the energy source, number, size, and substrates of the transporters, the EPs can be divided into five families (Li, X. Z. et al., 2016, chapter 6) that have been associated with drug resistance: the multidrug and toxic-compound extrusion (MATE) family and the small multidrug resistance (SMR) family, which are singlet transporters and occur in all species; and three superfamilies, which include the resistance nodulation and cell division (RND) super family, the ATP-binding cassette (ABC) superfamily, and the major facilitator superfamily (MFS), which are multicomponent transporters that operate through a complex efflux machinery that often include the pump, accessory adaptor proteins, and outer membrane proteins.

Bacterial EPs play a major role in the development of bacterial MDR. Efflux pump inhibitors (EPIs) maybe the best solution to this problem, as they can reverse resistance in bacteria (Li, X. Z. et al., 2016, chapter 29). EPIs act against MDR EPs with a wide substrate spectrum, and they are expected to not only reverse resistance to a single drug but also to multiple clinically beneficial antimicrobials at the same time. However, many discovered EPIs have either failed the preclinical trials, have failed to reach clinical concentrations for their activity *in vivo*, or have toxicity problems (Li, X. Z. et al., 2016, chapter 28). The effects of some EPIs on antimicrobial susceptibilities have been studied in several microorganisms, including *Escherichia coli* (Opperman et al., 2014), *Salmonella* (Sutkuviene et al., 2013), *Klebsiella pneumoniae* (Chevalier et al., 2004), *Mycobacterium tuberculosis* (Coelho et al., 2015; Machado et al., 2017), *Acinetobacter baumannii* (Richmond et al., 2013), and *Campylobacter* (Kurincic et al., 2012). However, in regard to this, no systematic, comprehensive research has been conducted in *R. anatipestifer*.

In this study, we used the standard broth microdilution method to determine the antibiotic susceptibility of a wide range of antibiotics against *R. anatipestifer* isolates, collected from different regions of China between 2010 and 2017. The presence of resistance genes and integrons in *R. anatipestifer* were detected by PCR. Through comparative analysis of a resistant phenotype and genotype of *R. anatipestifer* strains, we determined the extent of resistance in some *R. anatipestifer* isolates toward antimicrobial agents. Characterization of the arrays of integrons is a useful epidemiological tool in the study of the evolution of MDR. Various functions, especially drug resistance, were mediated into hosts *via* mobile gene cassettes through specific excision and integration in integrons (van Essen-Zandbergen et al., 2007; Li Y. et al., 2018). For *R. anatipestifer*, five resistance gene cassettes (*dhfrI–aadA2, aadA11, aadA5, aa6II–catB3–aadA1*, and *aadA1–dhfrA1*) of class 1 integrons have been reported (Zheng et al., 2012). The resistance observed could not be explained solely by the presence of the drug-resistant genes or mutations (Jaillard et al., 2017; Uddin and Ahn, 2017). Some *R. anatipestifer* isolates, in which drug-resistant genes were not detected, still showed resistance and some even showed MDR. These findings led us to explore the activity of EPs and its association with drug resistance.

We chose two broad-spectrum EPIs: carbonyl cyanide m-chlorophenylhydrazone (CCCP; Li Y. F. et al., 2016) that acts as an energy blocker and can inhibit EPs that are driven by hydrogen ion gradients; and Phe-Arg-β-naphthylamide (PAβN; Lamers et al., 2013), a protonophore inhibitor that can affect membrane integrity. These EPIs perturb the bacterial efflux systems, such as MFS and RND. In this study, we presume that MFS or RND EPs may play a major role in resistance associated with antibiotics in *R. anatipestifer* isolates.

The main purpose of this study was to assess the role played by the EP systems in the resistant phenotype and genotype of *R. anatipestifer* strains that carry different drug-resistant genes. In order to achieve this, we explored the contribution of the efflux mechanisms in the overall resistance to seven different types of antibiotics, in nine resistant clinical isolates of *R. anatipestifer* from four distinct geographical areas of China. The following processes were performed: (1) analysis of the synergistic effect of the efflux inhibitors, CCCP and PAβN, on the minimum inhibitory concentrations (MICs) of the antibiotics; (2) analysis of real time efflux activity to confirm the existence of active EP systems in these strains, using ethidium bromide (EB) as an efflux substrate, and subsequent calculation of final fluorescence both in the presence and absence of each efflux inhibitor; and (3) analysis of the levels of mRNA transcription (n-fold) of selected efflux pump genes (EPGs) in the strains under investigation by RT-qPCR. The results revealed that the addition of efflux inhibitors enhanced the effects of antibacterial drugs, independently of the genotypic resistance of the *R. anatipestifer* strains. The present study indicated the validity of a synergistic combination of drugs with traditional therapy, despite the presence of drug-resistant genes. This strategy proved to be beneficial, as it reduced the level of resistance among the strains. Moreover, the findings of the present study strongly supported the potential of these efflux inhibitors as adjuvants in the chemotherapy of *R. anatipestifer* infections, and these findings helped to increase the understanding of the MDR mechanisms of *R. anatipestifer*.

## Materials and methods

### Strains

The strains selected for this study are described in Table 1. All strains were preserved in the laboratory. The field isolate strains of *R. anatipestifer* (RA) were obtained from duck brain homogenates between March 2010 and December 2017, as previously described (Zheng et al., 2012). The samples were collected from diseased ducks in Shandong, Henan, Gongdong, and Anhui Provinces of China. The strains were categorized as multidrug resistant (resistant to at least three classes of antibiotics) and extensively drug resistant (MDR plus resistance to any fluoroquinolone and ampicillin, cefoxitin, or florfenicol) based on Table 2 data. The specific details of antimicrobial resistance phenotypes for each isolate are shown in Table 1. The RA01, ATCC11845, and *E. coli* ATCC25922 reference strains were obtained from the American Type Culture Collection (Virginia, USA) and were used as controls.

**Table 1:** Categorization of the *R. anatipestifer* strains that were studied according to their serotype, resistance pattern, and phenotype.

**Strain**	**Source**	**Serotype**	**Resistance pattern**	**Phenotype**
RA01	ATCC	6	None	Pan-Susceptible
RA70	Isolate	8	SMT	Monoresistant to SMT
HN352	Isolate	6	FLR	Monoresistantto FLR
RA66	Isolate	1	ROX	Monoresistant to ROX
HN313	Isolate	2	AMP	Monoresistant to AMP
HN333	Isolate	10	QUO	Resistant to QUO
FS8	Isolate	1	STR, KAN, GEN, SPE, TOB,SMT, TET, ROX	MDR
WF7	Isolate	2	STR, KAN, GEN, TOB, CIP, ENO, FLR, TET, OXT	MDR
GD01	Isolate	2	STR, KAN, GEN, SPE, AMK, TOB, NA, CIP, ENO, AMP, CEF, CHL, FLR, SMT, TET	XDR
SD314	Isolate	7	STR, KAN, GEN, SPE, AMK, NEO, TOB, NA, CIP, AMP, FLR, SMT, ROX, TET	XDR

*STR, streptomycin; KAN, kanamycin; GEN, gentamicin; SPE, spectinomycin; AMK, amikacin; NEO, neomycin; TOB, tobramycin; NA, nalidixic acid; CIP, ciprofloxacin; ENO, enrofloxacin; AMP, ampicillin; CEF, cefoxitin; CHL, chloramphenicol; FLR, florfenicol; SMT, sulfamonomethoxine; ROX, roxithromycin; TET, tetracycline; OXT, oxytetracycline; QUO, quinolones (NA, CIP, ENO). ATCC, American Type Culture Collection; Reference strains, RA01, ATCC11845; MDR, multidrug-resistance, resistant to at least three classes of antimicrobial agents; XDR, extensive drug-resistance, resistant to at least five classes of antimicrobial agents*.

**Table 2:** MIC of antibiotics and EB for the *R. anatipestifer* strains studied.

**Strain ID**	**MIC (**μ**g/mL)**																		
	**Antibiotics**																		**EB**
	**STR**	**KAN**	**GEN**	**SPE**	**AMK**	**NEO**	**TOB**	**NA**	**CIP**	**ENO**	**AMP**	**CEF**	**CHL**	**FLR**	**SMT**	**ROX**	**TET**	**OXT**	
Susceptible																			
RA01	8	4	2	2	2	8	2	4	0.125	0.125	1	0.25	0.5	0.25	8	0.125	0.25	0.5	0.6
SMT^R^																			
RA70	16	16	8	8	4	32	16	8	0.25	0.125	1	0.5	0.5	0.5	64	0.125	0.25	2	1.0
FLR^R^																			
HN352	16	16	16	32	32	32	8	8	0.25	0.5	4	0.25	8	16	32	2	2	2	1.0
ROX^R^																			
RA66	8	16	8	32	16	32	16	32	2	2	2	0.5	2	1	64	32	4	4	1.0
AMP^R^																			
HN313	32	32	16	64	8	64	32	32	1	1	16	2	2	0.5	16	16	4	2	1.0
QUO^R^																			
HN333	16	16	32	32	32	32	64	64	8	8	1	0.25	2	1	16	8	2	1	1.2
MDR																			
FS8	64	64	64	64	8	16	64	16	4	1	1	0.25	1	0.5	64	16	16	8	2.0
WF7	64	64	64	16	16	16	64	16	4	4	1	0.5	2	8	16	2	8	8	2.0
XDR																			
GD01	64	64	64	64	32	16	64	64	4	4	16	4	32	8	64	0.5	8	2	2.0
SD314	64	64	64	64	32	64	64	64	4	1	8	1	4	2	64	8	8	2	2.0

*STR, streptomycin; KAN, kanamycin; GEN, gentamicin; SPE, spectinomycin; AMK, amikacin; NEO, neomycin; TOB, tobramycin; NA, nalidixic acid; CIP, ciprofloxacin; ENO, enrofloxacin; AMP, ampicillin; CEF, cefoxitin; CHL, chloramphenicol; FLR, florfenicol; SMT, sulfamonomethoxine; ROX, roxithromycin; TET, tetracycline; OXT, oxytetracycline; EB, Ethidium bromide. Green represents sensitivity; red represents resistance; purple represents intermediate*.

### Drug susceptibility testing and MIC determination

The selection of antibiotics in the present study was made based on those most widely used in the duck industry (Zhong et al., 2009; Li Y. et al., 2016). These antibiotics were purchased from Solarbio (Beijing, China). The strains were tested to determine the MICs of 18 antibiotics (Table 2 and Table S1), according to the Clinical and Laboratory Standards Institute's (CLSI) 2-fold serial broth microdilution method (CLSI, 2016). Briefly, each *R. anatipestifer* isolate was seeded in trypticase soy broth (TSB) with 5% calf serum, shaken at 200 rpm (revolutions per minute) for 6–12 h, before being incubated at 37°C for 24 h with 5% CO_2_. A volume of 200 μL of the microbial suspension of 10^6^ CFU/mL was added to each well, each antimicrobial agent was added in successive rows, to attain concentrations between 64 and 0.125 μg/mL. All studies were carried out in triplicates. The MIC was recognized as the lowest concentration of the antimicrobial agent that can inhibit visible growth of bacteria (CLSI, 2016). The RA01, ATCC11845, and *E. coli* ATCC25922 strains were used as controls. The 18 antibiotics tested included β-lactams (ampicillin and cefoxitin); aminoglycosides (kanamycin, streptomycin, gentamicin, spectinomycin, amikacin, neomycin, and tobramycin); quinolones (nalidixic acid, ciprofloxacin, and enrofloxacin); amphenicols (chloramphenicol and florfenicol); sulfonamides (sulfamonomethoxine); macrolides (roxithromycin); and tetracyclines (tetracycline and oxytetracycline).

### Detection of resistance genes, mutations associated with resistance, and integrons

To prepare genomic DNA, the bacterial suspension was collected and heated at 100°C for 10 min and then incubated on ice for 10 min. The samples were then centrifuged (Thermo Pico17) at 13,800 × g for 5 min. The supernatants were collected and stored at 4°C until further use. The PCR amplification of quinolone resistance determining regions (QRDR); plasmid-mediated quinolone resistance (PMQR) genes; aminoglycoside resistance genes; beta-lactamase resistance genes; tetracycline resistance genes; amphenicol (chloramphenicol and florfenicol) resistance genes; sulfonamide resistance genes; *bla*_*NDM*−1_ and *mcr-1* resistance genes; class 1, 2, and 3 integrase genes; and a variable region of class 1, 2, and 3 integrons was carried out using the primers presented in the Supplementary Material (Table S2). The PCR amplification was carried out using a Takara Taq Master Mix Kit (TakaRa, Dalian, China) for standard PCR. The annealing temperature for each pair of primers is shown in Table S2. The positive PCR products were sequenced by the Nanjing Genscript Biology Company. Sequence data were then analyzed by DNASTAR and the sequences were compared with reference sequences from NCBI GenBank.

### Drug susceptibility testing of antibiotics in the presence and absence of efflux inhibitors

To determine the impact of active EP systems on resistance of *R. anatipestifer* isolates, a panel of nine *R. anatipestifer* drug-resistant strains was characterized for drug resistance genes and antibiotic profiles, in the presence and absence of PAβN and CCCP. The final use concentrations of the EPIs used were PAβN at 40 μg/mL and CCCP at 5 μg/mL. The correlation between the effect of the EPIs on the MICs of the antibiotics was assessed by synergism assays (refer to the previously mentioned broth microdilution method). The determination of MICs and the interpretation of results were performed as previously described (Sun et al., 2012). A reduction in MIC of at least 4-fold was considered as indicative of efflux (Li, X. Z. et al., 2016).

To quantify the role of the EPs in the strains under investigation and to establish a clinical association, we investigated the time taken for the detection of growth rate of each strain subjected to the antibiotics, in the presence and in the absence of the EPIs. The assays were performed as follows. Phe-Arg-β-naphthylamide (40 μg/mL) and CCCP (5 μg/mL) were added to the culture medium. These concentrations failed to affect the growth of the bacteria. Each antibiotic was tested at half MIC (determined for each strain), a concentration that does not affect the growth of *R. anatipestifer* isolates. All experiments were performed at least thrice for verification. Each time, bacterial strains were grown with TSB at 37°C until the cells reached an optical density (OD) at 600 nm of 1.0 (10^11^–10^12^ cfu/mL; Ji et al., 2017; Li S. et al., 2018). A 2% inoculum (100 μL in 5 mL) of bacterial culture was added to fresh TSB. This suspension was incubated under shaking conditions (200 rpm) at 37°C. The OD at a wavelength of 600 nm was measured over a period of 12 h using a UV spectrophotometer (Beckman Coulter, DU730, USA). The evaluation of bacterial growth *vs*. growth in the presence of antibiotics *vs*. growth in a mixture of antibiotics and efflux inhibitor was performed, by comparing the ODs of each strain, starting at the first hour and at 1 h intervals thereafter. Thus, the degree to which PAβN and CCCP enhanced the potentiation of antibiotic activity was determined.

### Ethidium bromide accumulation and efflux assay

For the standard EB accumulation assays, cells were grown to an OD_600_ of 1.0 (10^11^–10^12^ cfu/mL) and 200 μL of cells were loaded into 96-well black plates and mixed with EB for 60 min to determine EB accumulation. To evaluate the effects of the efflux inhibitors' impact on efflux activity based on the accumulation of EB, the EB-loaded cells were washed and resuspended in phosphate-buffered saline (PBS), either with or without the EPIs, PAβN (40 μg/mL) and CCCP (5 μg/mL). The plates were kept at room temperature for 15 min and then fluorescence detection was performed. Each assay lasted for 40 min, and the signal was acquired every 5 min. Specific EB concentrations are indicated in the legends of Figures S1, S2. Fluorescence was determined by a microplate reader (Varioskan LUX, Thermo Fisher, USA) at emission and excitation wavelengths of 605 and 525 nm, respectively (Liu et al., 2017). The raw data obtained was normalized by comparing the relative fluorescence units obtained under the conditions that promote efflux (in the presence of 5% calf serum and in the absence of efflux inhibitors) with the relative fluorescence units from the control, in which no efflux was evident (in the presence of efflux inhibitors with 5% calf serum). The raw data was also normalized to the EB-loaded cells to which 5% calf serum was added, as the relative fluorescence of these cells was considered to be equivalent to 1 (Machado et al., 2017).

### RT-qPCR analysis of putative efflux pump genes

Strains were grown to an OD_600_ of 1.0 (10^11^–10^12^ cfu/mL) in shake flasks containing TSB, 5% calf serum, and half the MIC of each antibiotic as follows: RA70 was exposed to sulfamethazine (SMT); HN352 was exposed to amikacin (AMK); RA66 was exposed to roxithromycin (ROX); HN313 was exposed to ampicillin (AMP); HN333 was exposed to QUO (ciprofloxacin or enrofloxacin); FS8 was exposed separately to SMT, ROX, and ciprofloxacin (CIP); WF7 was exposed separately to florfenicol (FLR), CIP, and oxytetracycline (OXT); GD01 was exposed separately to FLR, AMP, CIP, AMK, and chloramphenicol (CHL); SD314 was exposed separately to FLR, ROX, AMP, CIP, neomycin (NEO), and AMK. The antibiotics were chosen for testing based on the MIC changes for each strain, which was affected by the EPIs. Although there are many factors that promote drug resistance, such as drug inactivating enzymes, alteration of drug targets, mobile resistant genetic elements; in this study, since the MICs of the antibiotics were reduced in the presence of EPIs by at least 4-fold, this was considered indicative of efflux activity (see Tables 2, **4**, Table S1).

Moreover, to evaluate the effects of the antibiotics alone and in combination with the efflux inhibitors on EP activity, we analyzed the expression of the EPGs exposed to the antibiotic alone or combined with the inhibitors PAβN (40 μg/mL) and CCCP (5 μg/mL). The relative expression of the EPGs was assessed by comparing the relative quantity of the respective mRNA in the presence of the antibiotic, and the antibiotic + sub-MIC concentration of the inhibitor, with those of the non-exposed strain. Total RNA was extracted using Invitrogen™ Trizol™ Max™ Bacterial RNA Isolation Kit (Thermo Fisher Scientific, USA). The relative expression of the EPGs *RIA-1800, RIA-1853, RIA-0245, RIA-0257, RIA-0437, RIA-0577, RIA-0746, RIA-1554, RIA-1117, RIA-1118, RIA-1215, RIA-1993, RIA-0286, RIA-1069*, and *RIA-1614* was analyzed by RT-qPCR. The primers used for RT-qPCR are listed in the Supplementary Material (Table S3). Reverse transcription of complementary DNAs and quantitative reverse transcription-PCR were performed simultaneously, using an AceQ qPCR SYBR Green Master Mix Kit (Vazyme, Nanjing, China), a HiScript Q RT SuperMix for qPCR Kit (Vazyme, Nanjing, China), and a MX3000P instrument (Agilent Technologies, Santa Clara, USA).

The protocol that was used comprised the following amplification program: reverse transcription for 15 min at 50°C, followed by 2 min at 85°C, an initial denaturation step for 5 min at 95°C, and 40 cycles (95°C for 10 s and 60°C for 30 s). The specificity of all the primers was analyzed by using melting curves. The melting curves were recorded after the 40th cycle by increasing the temperature stepwise by 0.5°C every 5 s from 65 to 95°C. The comparative quantification cycle method was used to determine the relative mRNA expression of the efflux genes (Livak and Schmittgen, 2001). The relative expression of each target gene was determined by comparing the relative quantity of the respective mRNA in the presence of the antibiotic and in the presence of the antibiotic and efflux inhibitor to that of the non-exposed mRNA. Each strain was assayed in triplicates, using the RNA obtained from three independent cultures. A relative expression level equal to 1 indicated that the expression level was identical to the unexposed strain. Each sample cycle threshold (CT) mean was calculated and standard deviations were calculated for each mean CT value. Normalization of the mRNA levels was done using the *R. anatipestifer recA* housekeeping gene (Huang et al., 2017) as internal control for each experiment and presented as the mean-fold change (±SD) compared with the control. Furthermore, the 2^−ΔΔ*Ct*^ formula was used to calculate the relative expression levels of the efflux genes. The real time PCR data were detected and analyzed by the MxPro Software (Mx3000p Quantitative PCR) according to default parameters, which generated the CT values for each reaction.

### Ethics statement

All animal were handled by following strict procedures according to the (indicate as appropriate). The study was approved by the Institutional Animal Care and Use Committee of the Lanzhou Veterinary Research Institute.

### Statistical analysis

The data were analyzed using two-way ANOVA multiple comparisons using the GraphPad Prism 6.0 Windows software. Levels of significance were set as follows: ^*^P < 0.05 was considered statistically significant, and ^**^P < 0.01, ^***^P < 0.001, and ^****^P < 0.0001 were all considered highly significant.

## Results

### Correlating phenotype and genotype associated with antibiotic resistance

To establish associations among phenotypic drug resistance and resistance determinants, we searched for the presence of resistance genes, mutations in resistance genes, and integrons associated specifically with resistance to the antibiotics under investigation.

First, according to the MICs of each antibiotic (Table 2), 10 *R. anatipestifer* isolates from six different serotypes were selected, based on their drug resistance patterns (Table 1). The distribution of MIC values for each antibiotic is shown in Table 2. Multidrug-resistant isolates were resistant to at least three antimicrobial agents, and extensively drug-resistant isolates were multidrug-resistant strains that were additionally resistant to any fluoroquinolone and ampicillin, cefoxitin, or florfenicol. The *R. anatipestifer* isolates exhibited resistance to many antimicrobial agents, particularly aminoglycosides, fluoroquinolones, and sulfonamides, all of which are commonly used in ducks.

The distribution of resistance genes, integrons, and QRDR mutations is shown in Table 3. Among the aminoglycoside resistance genes, eight aminoglycoside modifying enzyme genes were identified. The *aac(6*′*)-Ib* gene that causes resistance to kanamycin, tobramycin, and amikacin was detected in all the isolates. This is the most common resistance gene found in clinical isolates that were resistant to aminoglycosides. The *aadA1, aadA2*, and *aadA5* genes that confer resistance to spectinomycin and streptomycin, the *aadA1* and *aadA2* were detected in almost all of the isolates, but the *aadA5* gene was detected in the HN333 and SD314 isolates only. The *aac(3*′*)-IIc* gene that causes resistance to gentamicin and tobramycin was detected in seven isolates. The *aac(3*′*)-IV* gene that mediates resistance to tobramycin, gentamicin, and neomycin was detected in the HN352, GD01, and SD314 isolates. The *aph(3*′*)-VII* and *aph(2*′*)-Ib* genes that cause resistance to amikacin, neomycin, and kanamycin were detected in the HN352, HN333, GD01, and SD314 isolates. Among the macrolide-resistance genes, only the *emrF* gene was detected in five isolates. Among the chloramphenicol- and florfenicol-resistance genes, *cat2, cmlA*, and *floR* were detected in the WF7, GD01, and SD314 *R. anatipestifer* isolates, respectively. Among the tetracycline and sulfonamide resistance genes, *tet(*A*), tet(*B*), sul1, sul2*, and *sul3* were present in the RA70, RA66, HN313, FS8, WF7, GD01, and SD314 isolates. Among the beta-lactamase resistance genes, the *bla*_*TEM*_ gene was detected in the HN313, HN333, FS8, and GD01 isolates. Only the *bla*_*OXA*_gene was detected in the SD314 isolate. One 16S rRNA methylase gene (*rmtD*) was detected in the HN333 isolate. Notably, two resistance genes (*bla*_*NDM*−1_and *mcr-1*) were not detected. Eight *R. anatipestifer* isolates harbored one or two class 1 or class 2 integrons. Four different cassette arrays (*aadA1, aadA2, aadA5*, and *aacA4-aadA1*) of the class 1 integron and the *sat2-aadA1* gene cassette of the class 2 integron were discovered. The class 3 integron was not detected in the strains under investigation. The *aadA1* and *aac(6*′*)-Ib-floR* gene cassettes that cause resistance to aminoglycoside antibiotics and chloramphenicol were detected in the WF7, GD01, and SD314 isolates.

**Table 3:** Characteristics of drug-resistant genes of *R. anatipestifer* isolates used in this study.

**Strain ID**	**Resistance genes**	**Class 1 integron gene cassette**	**Class 2 integron gene cassette**	**QRDR Mutations**				**PMQR**
				***gyrA***	***gyrB***	***parC***	***parE***	
Susceptible								
RA01	*aac(6′)-Ib*	–	–	WT	WT	WT	WT	–
SMT^R^								
RA70	*aadA1, aac(6′)-Ib,aac(3′)-IIc, sul1, sul3*	*aadA1*	–	WT	WT	WT	WT	–
FLR^R^								
HN352	*aadA1, aac(3′)-IV, aac(6′)-Ib, aac(3′)-IIc, tet(A)*	*aadA1*	–	Ser83Ile	WT	WT	WT	–
ROX^R^								
RA66	*aadA2, aac(6′)-Ib, tet(A), emrF*	*aadA2*	–	WT	WT	WT	WT	–
AMP^R^								
HN313	*aadA2, aac(3′)-IIc, aac(6′)-Ib, bla_*TEM*_, tet(B)*	–	–	WT	WT	WT	WT	–
QUO^R^								
HN333	*aadA5, aph(3′)-VII, aac(6′)-Ib, rmtD, bla_*TEM*_,emrF*	*aadA5*	–	Ser83Ile	Ser121Phe	Gly313Ser	WT	*qnrS*
MDR								
FS8	*aadA1, aac(6′)-Ib, bla_*TEM*_, tet(A), emrF, sul2, sul3*	–	*sat2—aadA1*	Asp87His	WT	WT	WT	–
WF7	*aadA1, aac(3′)-IIc, aac(6′)-Ib, cat2, floR, tet(A), tet(B)*	*aadA1*	*sat2—aadA1*	Ser83Ile	WT	Gly313Ser	WT	*qnrD*
XDR								
GD01	*aacA4–aadA1, aac(3′)-IIc,aph(3′)-VII, aac(3′)-IV, bla_*TEM*_, aac(6′)-Ib, tet(A), cmlA, floR, emrF, sul3*	*aacA4—aadA1*	–	Ser83Ile	WT	WT	WT	–
SD314	*aadA1, aadA5, aac(3′)-IV, aac(6′)-Ib, aph(3′)-VII, aph(2′)-Ib, bla_*TEM*_, bla_*OXA*_, cat2, floR, sul3*	*aadA5*	–	Ser83Arg	WT	Gly313Ser	WT	*qnrS*

*SMT, sulfamonomethoxine; AMK, amikacin; ROX, roxithromycin; AMP, ampicillin; QUO, quinolones (NA, CIP, ENO). MDR, multidrug-resistance; XDR, extensive drug-resistance. WT, wild type sequence. QRDR, quinolone resistance determining regions; PMQR, plasmid-mediated quinolone resistance*.

Finally, in light of the MICs of quinolones and fluoroquinolones, ten *R. anatipestifer* isolates were selected for the detection of QRDR mutations (*gyrA, gyrB, parC*, and *parE*) and PMQR. The distribution of the QRDR gene mutations and PMQR genes are listed in Table 3. Four isolates exhibited the wild type genes *gyrA, gyrB, parC*, and *parE*. Three isolates had a single mutation in *gyrA* at codon 83 (Ser to Ile) or codon 87 (Asp to His). Two isolates had one *parC* mutation (Gly313 to Ser) and one *gyrA* hotspot mutation. One isolate had one *gyrB* mutation (Ser121 to Phe), one *parC* mutation (Gly313 to Ser), and one *gyrA* hotspot mutation. No mutations were detected in the *parE* genes. In regard to the detection of PMQR genes, three isolates were observed to carry two *qnrS* genes and one *qnrD* gene, respectively. The *R. anatipestifer* isolates, particularly HN333, GD01, and SD314, were resistant to quinolones in the broth microdilution assay at the highest concentrations (Table 2).

Briefly, 18 antibiotics out of the seven classes of antibiotics were evaluated, and we selected one representative antibiotic family for a subsequent study. For example, the resistance phenotype detected was mostly consistent with the detection of resistance genes among the majority of isolates, such as the *aac(6*′*)-Ib* or *aph(3*′*)-VII* gene. These genes were associated with amikacin and kanamycin resistance. The QRDR mutation and PMQR, which are quinolone resistance genes, were tested in all *R. anatipestifer* strains; the HN352 isolate carrying *gyrA* (Ser83 to Ile) was susceptible to quinolone, and FS8 isolate that was negative for PMQR was resistant to quinolones. The *bla*_*TEM*_ gene confers ampicillin resistance and *aac(6*′*)-Ib* is an amikacin resistant gene, whereas the WF7 isolate that carries the *aac(6*′*)-Ib* gene was susceptible to amikacin, and SD314 isolates negative for *bla*_*TEM*_ were resistant to ampicillin. Therefore, other resistance mechanisms may exist in *R. anatipestifer*.

### Quantification of antibiotic resistance in the presence of efflux pump inhibitors

We quantified the MICs of antibiotics for specific strains in the presence or absence of EPIs. To achieve this, we selected representative clinical strains of *R. anatipestifer* from four distinct geographical areas. The MICs of the antimicrobial agents, both in the presence and absence of EPIs, were determined for each strain and the results are presented in Table 4. The MICs observed in the presence or absence of EPIs showed considerable differences among the seven groups of strains. Similarly, the MICs of the specific antimicrobial agents showed different MIC values. Therefore, we assumed that the different resistance levels observed for the same genotype or phenotype were due to the different levels of active efflux of the antibiotics. The existence of active efflux was evaluated through the reduction of the MICs for the antibiotics in the presence of the EPIs CCCP and PAβN, growth rate analyses, and EP gene expression.

**Table 4:** Quantitative drug susceptibility testing in the presence and absence of efflux inhibitors.

**Antibiotic**		**Quantitative susceptibility testing (MIC** μg/mL)*								
		**SMT^R^**	**FLR^R^**	**ROX^R^**	**AMP^R^**	**QUO^R^**	**MDR**		**XDR**	
		**RA70**	**HN352**	**RA66**	**HN313**	**HN333**	**FS8**	**WF7**	**GD01**	**SD314**
SMT	No EI	64	–	–	–	–	64	–	–	–
	+CCCP	32	–	–	–	–	16	–	–	–
	+PAβN	8	–	–	–	–	4	–	–	–
FLR	No EI	–	16	–	–	–	–	8	8	2
	+CCCP	–	0.25	–	–	–	–	0.5	0.25	0.25
	+PAβN	–	0.5	–	–	–	–	1	1	1
ROX	No EI	–	–	32	–	–	16	–	–	8
	+CCCP	–	–	2	–	–	2	–	–	1
	+PAβN	–	–	8	–	–	4	–	–	4
AMP	No EI	–	–	–	16	–	–	–	16	8
	+CCCP	–	–	–	4	–	–	–	16	8
	+PAβN	–	–	–	1	–	–	–	1	0.25
CIP	No EI	–	–	–	–	8	4	4	4	4
	+CCCP	–	–	–	–	4	4	4	1	4
	+PAβN	–	–	–	–	2	1	0.125	0.125	1
ENO	No EI	–	–	–	–	8	–	–	–	–
	+CCCP	–	–	–	–	0.5	–	–	–	–
	+PAβN	–	–	–	–	0.5	–	–	–	–
OXT	No EI	–	–	–	–	–	–	8	–	–
	+CCCP	–	–	–	–	–	–	0.5	–	–
	+PAβN	–	–	–	–	–	–	4	–	–
CHL	No EI	–	–	–	–	–	–	–	32	–
	+CCCP	–	–	–	–	–	–	–	2	–
	+PAβN	–	–	–	–	–	–	–	8	–
AMK	No EI	–	–	–	–	–	–	–	32	32
	+CCCP	–	–	–	–	–	–	–	1	2
	+PAβN	–	–	–	–	–	–	–	8	16
NEO	No EI	–	–	–	–	–	–	–	–	64
	+CCCP	–	–	–	–	–	–	–	–	4
	+PAβN	–	–	–	–	–	–	–	–	32

SMT, sulfamonomethoxine; FLR, florfenicol; ROX, roxithromycin; AMP, ampicillin; CIP, ciprofloxacin; ENO, enrofloxacin; OXT, oxytetracycline; CHL, chloramphenico; AMK, amikacin; NEO, neomycin; QUO, quinolones (NA, CIP, ENO). *The lowest concentration tested corresponded to the critical concentration for each antibiotic (see section Materials and Methods for details). MDR, multidrug-resistance; XDR, extensive drug-resistance. CCCP (5μg/mL), carbonyl cyanide-m-chlorophenylhydrazone; PAβN (40μg/mL), Phe-Arg-β-naphthylamide.

Table 4 illustrates the strains that showed MIC values of the antibiotics with a reduction of 4-fold or above in the combination of CCCP and PAβN. The RA70 and FS8 strains that were resistant to SMT showed high level resistance (*R* = 64 μg/mL). The addition of PAβN reduced the MIC of SMT (8- to 16-fold) and the addition of CCCP also reduced the MIC of SMT (2- to 4-fold).

For HN352, WF7, GD01, and SD314, the MIC for FLR (*R* ≥ 2–16 μg/mL) was reduced more than 8-fold by the pump inhibitors. Regarding ROX resistance (*R* ≥ 8–32 μg/mL), the MIC values were reduced in the presence of CCCP in all ROX-resistant strains by 16-fold (RA66), 8-fold (FS8), and 8-fold (SD314); and in the presence of PAβN in all ROX-resistant strains by 4-fold (RA66), 4-fold (FS8), and 2-fold (SD314). The addition of PAβN was able to reduce the MIC of AMP (*R* ≥ 8–16 μg/mL) in HN313 (16-fold), GD01 (16-fold), and SD314 (32-fold). In contrast, the addition of CCCP failed to modify the MIC of AMP for GD01 and SD314; the same trend was not observed in HN313. Of the five strains (Table 4) resistant to CIP (*R* ≥ 4–8 μg/mL), with the exception of GD01 (with a 4-fold reduction), none were reduced in the presence of the CCCP inhibitor. However, in the CIP-resistant strains, the MIC values were reduced from 4- to 32-fold, depending on the strains. Resistance to ENO (*R* = 8 μg/mL) was reduced in strain HN333 alone, by 16-fold in the presence of CCCP and PAβN. The MIC for OXT (*R* = 8 μg/mL) in the WF7 strain was reduced by 16-fold in the presence of CCCP alone. Similar to GD01, the MIC for CHL (R ≥ 32 μg/mL) was reduced by 16-fold in the presence of CCCP. The effect of the PAβN inhibitor on the WF7 and GD01 strains was not obvious.

With regard to resistance to AMK (*R* = 32 μg/mL), the results showed that the addition of CCCP reduced the MIC of GD01 (32-fold) and SD314 (32-fold), and, to a lesser extent, PAβN also reduced the MIC of GD01 (4-fold) and SD314 (2-fold). Similarly, the MIC of NEO (*R* = 64 μg/mL) in the SD314 strain was reduced by 16-fold in the presence of CCCP and by 2-fold in the presence of PAβN. These data indicated the presence of efflux systems that are associated with drug resistance in *R. anatipestifer*.

### Determination of the intrinsic efflux capacity of *R. anatipestifer* strains

Resistance, particularly MDR, takes many forms and is often a complex process. However, the primary response of most pathogens to antibiotic exposure requires a drug EP. A better understanding of the processes that are involved in studies that are more extensive would lead to improved treatment applications. To confirm the existence of active efflux systems in the strains under investigation, we assessed their ability to efflux EB by real time fluorometry (Viveiros et al., 2010; Amaral et al., 2011). To perform these assays, one representative from each drug-resistant group was selected as follows: RA70, mono-resistant to SMT; HN352, mono-resistant to FLR; RA66, mono-resistant to ROX; HN313, mono-resistant to AMP; HN333, resistant to QUO; FS8 and WF7, multidrug-resistant; and GD01 and SD314, extensively drug-resistant (Figures S1, S2). The *R. anatipestifer* strain RA01 was considered representative of drug-susceptible strains.

First, the lowest concentration that caused minimal accumulation of EB was determined. The lowest concentration that resulted in equilibrium between the influx and efflux of EB was 0.3 μg/mL for the reference strain RA01; 0.5 μg/mL for RA70, HN352, RA66, and HN313 mono-resistant strains; 0.6 μg/mL for the HN333 strain; and 1.0 μg/mL for the multidrug-resistant and extensively drug-resistant strains. These results indicated that the clinical antibiotic resistant *R. anatipestifer* strains could handle higher concentrations of EB than the reference strain and suggested the presence of more active EP systems in resistant strains. Figures S1, S2 illustrate the effects of the efflux of EB with different efflux activity efficiencies. The efflux of EB was also affected by the presence of 5% calf serum (compare the black dotted line with the colored curve on each graph), demonstrating that sufficient external nutrition of the cells was necessary to guarantee optimal efflux activity in *R. anatipestifer*. The assays showed that all strains under investigation presented efflux activity. This is an intrinsic characteristic of susceptible and drug-resistant strains of *R. anatipestifer*.

The varied abilities for the efflux of EB demonstrated by the strains were observed as follows: RA01 presented basal efflux activity; in the clinical GD01 and WF7 strains (Figure S2), efflux activity was more pronounced; the EB efflux activity of HN352, RA66, HN313, FS8, and SD314 strains was significant and has similar levels, which was less than that of the GD01 and WF7 strains; and RA70 (Figure S1) and HN333 (Figure S2) presented diverse efflux activity. In most of the strains, the efflux inhibitor that yielded the highest inhibitory effect was CCCP, followed by PAβN; however, in HN333, the highest EB accumulation rate was achieved following the addition of PAβN, as assessed by the fluorometric method. These results might be associated with the different environmental conditions and drugs to which these strains were subjected to in the clinical setting. The results also revealed that the two inhibitors were able to reduce real time efflux activity in the majority of the strains and provided further evidence that active efflux was inhibited by these compounds.

### Effects of antibiotics and efflux inhibitors on efflux pump gene expression

**Figure 1:**
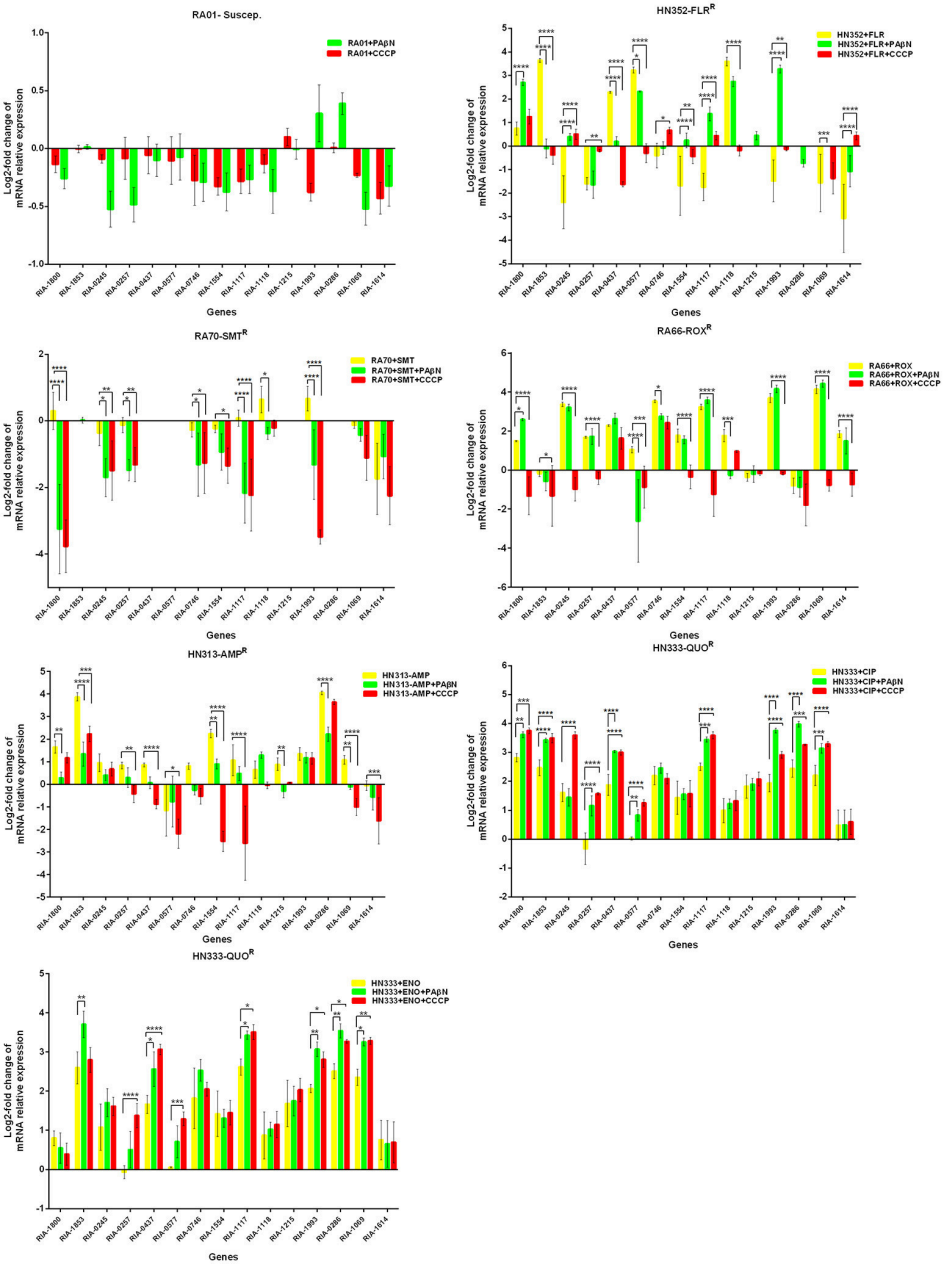
Quantification of the relative mRNA expression levels of a panel of EPGs in mono-resistant strains. Strains were grown in the presence of half MIC of each antibiotic; CCCP (5 μg/mL) and PAβN (40 μg/mL), showed no effects on the growth of *R. anatipestifer* isolates. The MICs of the antibiotics and EPIs were determined for each strain and are presented in Table 4. Change in the level of transcription of EPGs was measured as fold change normalized to *recA* gene expression and was subsequently calculated as log2-fold change that is relative to the untreated cell culture. Levels of significance were set as follows: *P < 0.05 was considered statistically significant and **P < 0.01, ***P < 0.001, and ****P < 0.0001 were all considered highly significant.

**Figure 2:**
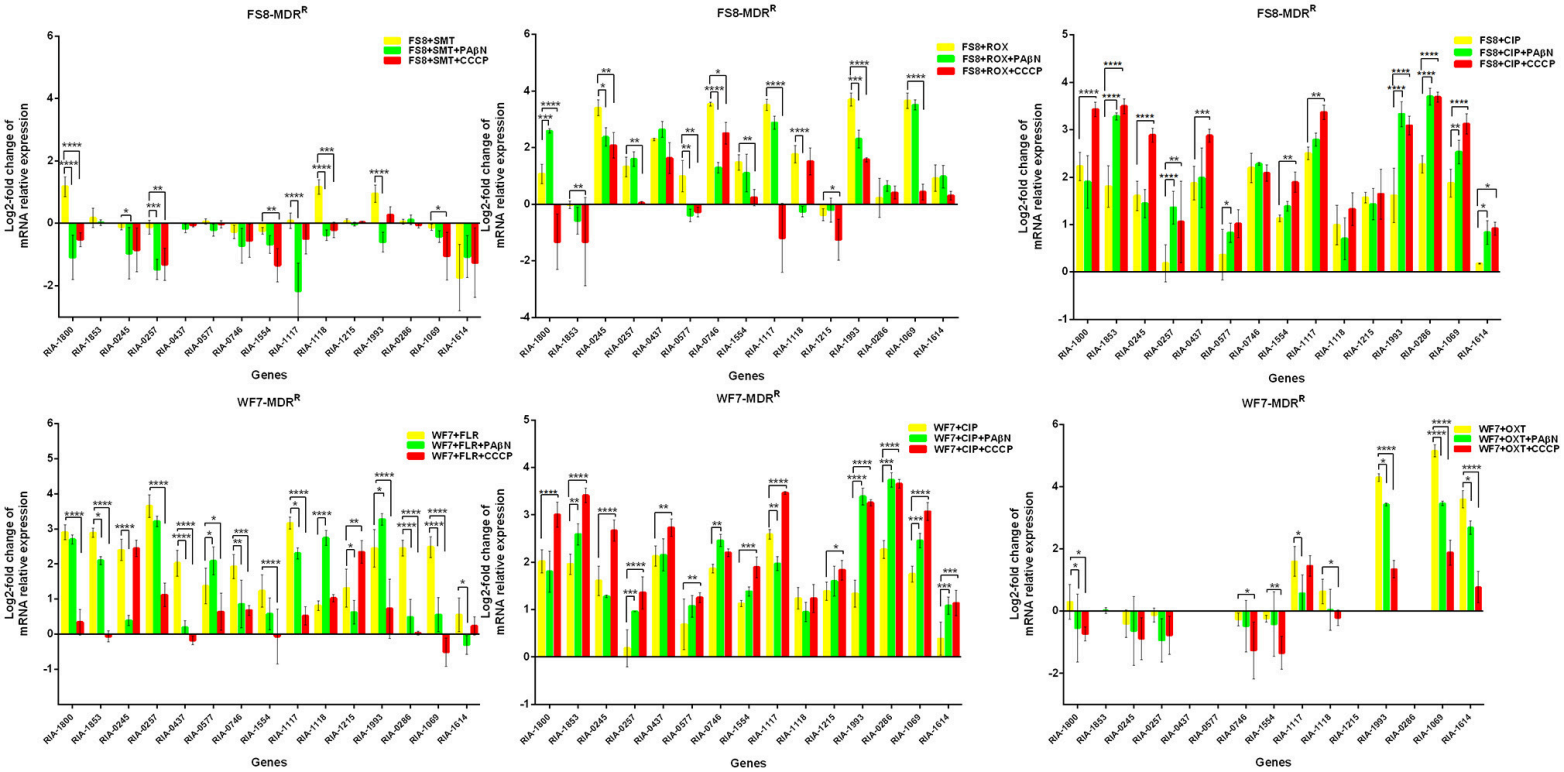
Quantification of the relative mRNA expression levels of a panel of EPGs in multidrug-resistant strains. Strains were grown in the presence of half MIC of each antibiotic; CCCP (5 μg/mL) and PAβN (40 μg/mL) showed no effects on the growth of *R. anatipestifer* isolates. The MICs of the antibiotics and EPIs were determined for each strain and are presented in Table 4. Change in the level of transcription of EPGs was measured as fold change normalized to *recA* gene expression and was subsequently calculated as log2-fold change that is relative to the untreated cell culture. Levels of significance were set as follows: *P < 0.05 was considered statistically significant, and **P < 0.01, ***P < 0.001, and ****P < 0.0001 were all considered highly significant.

**Figure 3:**
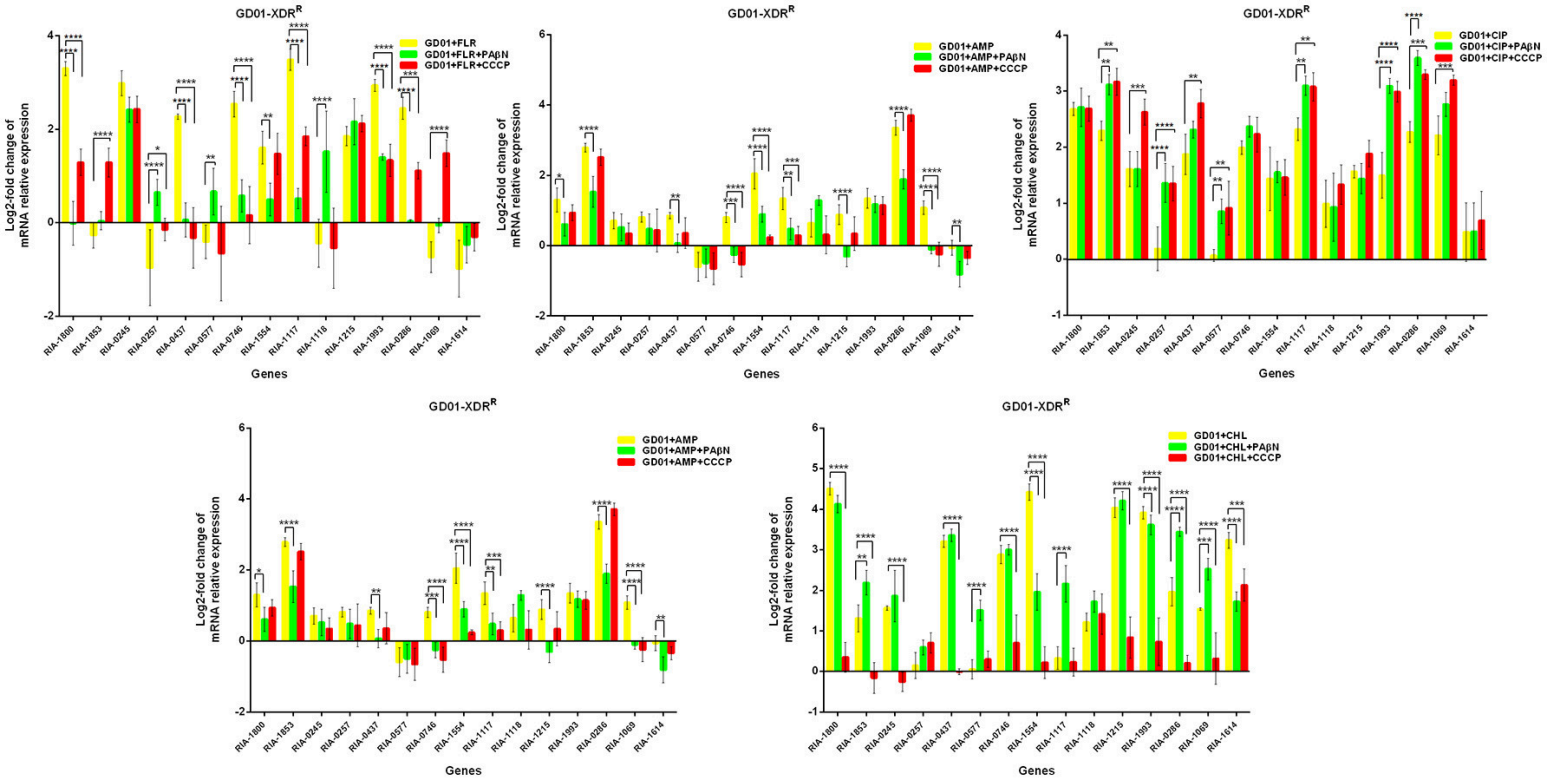
Quantification of the relative mRNA expression levels of a panel of EPGs in GD01 strains. Strains were grown in the presence of half MIC of each antibiotic; CCCP (5 μg/mL) and PAβN (40 μg/mL) showed no effects on the growth of *R. anatipestifer* isolates. The MICs of the antibiotics and EPIs were determined for each strain and are presented in Table 4. Change in the level of transcription of EPGs was measured as fold change normalized to *recA* gene expression and was subsequently calculated as log2-fold change that is relative to the untreated cell culture. Levels of significance were set as follows: *P < 0.05 was considered statistically significant, and **P < 0.01, ***P < 0.001, and ****P < 0.0001, were all considered highly significant.

**Figure 4:**
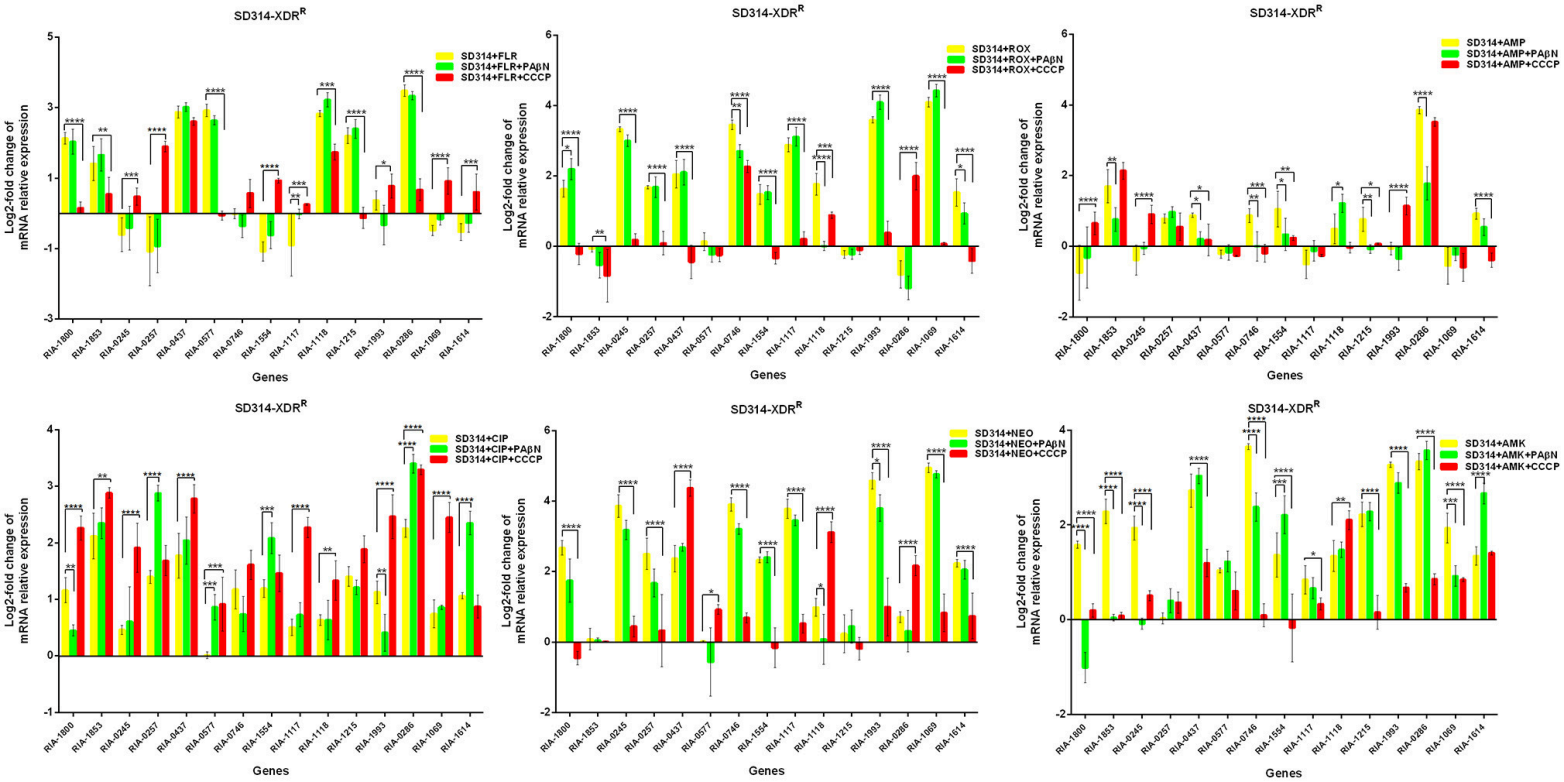
Quantification of the relative mRNA expression levels of a panel of EPGs in SD314 strains. Strains were grown in the presence of half MIC of each antibiotic; CCCP (5 μg/mL) and PAβN (40 μg/mL) showed no effects on the growth of *R. anatipestifer* isolates. The MICs of the antibiotics and EPIs were determined for each strain and are presented in Table 4. Change in the level of transcription of EPGs was measured as fold change normalized to *recA* gene expression and was subsequently calculated as log2-fold change that is relative to the untreated cell culture. Levels of significance were set as follows: *P < 0.05 was considered statistically significant, and **P < 0.01, ***P < 0.001, and ****P < 0.0001 were all considered highly significant.

Putative EPGs were selected by searching for genes in the National Center for Biotechnology Information (NCBI) database (Wang et al., 2012, 2015; Song et al., 2016), using combinations of the following keywords: “efflux pump,” “*R. anatipestifer*,” “MFS,” “RND,” “SMR,” “MATE,” and “ABC.” In addition, a review of previous articles led to the selection of 15 putative genes for further analysis in the present study (Table S4). To validate our previous findings, we analyzed the contribution of EPGs to antibiotic resistance in the selected strains. The quantification of the EP mRNA levels of the *R. anatipestifer* clinical isolates is shown in Figures 1–**4**.

With regard to the mono-resistant strains, when RA70 was exposed to SMT, a relatively small increase in the mRNA levels was observed for all the genes under investigation. When PAβN or CCCP was added, a significant reduction in the expression of four genes was evident, namely *RIA-1800, RIA-1117*, and *RIA-1993*. When the florfenicol-resistant strain, HN352 was exposed to FLR, among all the genes under investigation, four were observed to be overexpressed, namely *RIA-1853, RIA-0437, RIA-0577*, and *RIA-1118*. The addition of efflux inhibitors highlighted the contribution of different classes of EPs to different efflux levels in the various isolates used in the study. The presence of CCCP led to significantly reduced efflux in HN352 (with the exception of *RIA-0245, RIA-1117*, and *RIA-1614*). However, the addition of PAβN caused the greatest increase in the expression of *RIA-1800, RIA-1117*, and *RIA-1993*. Generally, the increase observed was significantly greater in the presence of CCCP (Figure 1).

In the case of RA66 exposed to ROX, the addition of PAβN and ROX highlighted the contribution of different classes of EPs to almost similar efflux levels that were observed in the HN352 isolates. In comparison to PAβN, CCCP caused a significant reduction in the efflux of RA66, in the following genes: *RIA-1800, RIA-0245, RIA-0257, RIA-0577, RIA-1554, RIA-1117, RIA-1993, RIA-1069*, and *RIA-1614*. In the HN313 strain exposed to AMP, five genes were found to be overexpressed, namely *RIA-1800, RIA-1853, RIA-1554, RIA-1117, RIA-0286*, and *RIA-1069*. The addition of CCCP caused a significant reduction in the expression of *RIA-1554, RIA-1117*, and *RIA-1069*. However, the addition of PAβN also caused a significant reduction in the expression of *RIA-1800, RIA-1853*, and *RIA-0286*. The QUO-resistant strain HN333, when exposed to CIP, showed significant overexpression of all the genes under investigation, with the exception of *RIA-0257, RIA-0577*, and *RIA-1614*. When exposed to enrofloxacin, HN333 showed significant overexpression of all the genes tested, with the exception of *RIA-1800, RIA-0257, RIA-0577*, and *RIA-1614*. In contrast, the addition of PAβN and CCCP caused a significant increase in the expression of some EPGs, such as *RIA-1853, RIA-0437, RIA-1117, RIA-1993, RIA-0286*, and *RIA-1069* (Figure 1). The multidrug-resistant FS8 strains were exposed to SMT, ROX, and CIP, respectively, and similar patterns were observed among the mono-resistant strains. Similarly, the multidrug-resistant WF7 strains were exposed to FLR or CIP, and similar patterns were observed among the mono-resistant strains. Three out of 15 genes were overexpressed upon exposure to OXT; *RIA-1069*, particularly, showed the highest level of expression in WF7 strains. The addition of PAβN and CCCP to the multidrug-resistant FS8 and WF7 strains showed similar effects (Figure 2). The extensively drug-resistant GD01 (Figure 3) and SD314 (Figure 4) strains that were exposed to various antibiotics showed different efflux levels. Overall, *RIA-1800, RIA-1118*, and *RIA-1993* were overexpressed in RA70 and FS8, independently of the SMT to which they were exposed. The *RIA-0437* gene was overexpressed in HN352, WF7, GD01, and SD314 strains, independently of the amphenicols to which it was exposed. The *RIA-0245, RIA-0746, RIA-1117, RIA-1993*, and *RIA-1069* genes were overexpressed in the RA66, FS8, and SD314 strains, independently of the ROX to which they were exposed. In addition, the *RIA-1853, RIA-1554*, and *RIA-0286* genes were overexpressed in the HN313, GD01, and SD314 strains, independently of the AMP to which they were exposed. In contrast, *RIA-0257, RIA-0577*, and *RIA-1614* showed the lowest levels of expression in ciprofloxacin-resistant strains and in response to ciprofloxacin antibiotics. When the extensively drug-resistant strains GD01 and SD314 were exposed to AMK, nine EPGs were found to be overexpressed. This suggested that the EPs of clinical isolates might be expressed at different levels in the presence of different antibiotics. To summarize, the addition of PAβN with various antibiotics affected the expression levels of EPs in a way similar to that observed with CCCP. Nevertheless, with the exception of QUO and AMP antibiotics, the reduction observed in expression following the addition of PAβN was not as high as that observed with CCCP.

### Evaluation of growth kinetics of the synergistic effect between efflux inhibitors and antibiotics

According to previous reports, the combination of an antibiotic and an efflux inhibitor at subminimal inhibitory concentrations delays the growth of *M. tuberculosis* drug-resistant strains (Coelho et al., 2015; Machado et al., 2017). Therefore, in *R. anatipestifer* drug-resistant strains, the EP is also believed to contribute to the overall level of antibiotic resistance described above. Furthermore, the delay in the growth of each drug-resistant strain, due to the stress imposed by the combination of an antibiotic and an efflux inhibitor at subminimal inhibitory concentrations, will render them more susceptible to the effects of the antibiotic. To test this hypothesis, we conducted drug susceptibility tests for the selected *R. anatipestifer* strains.

**Figure 5:**
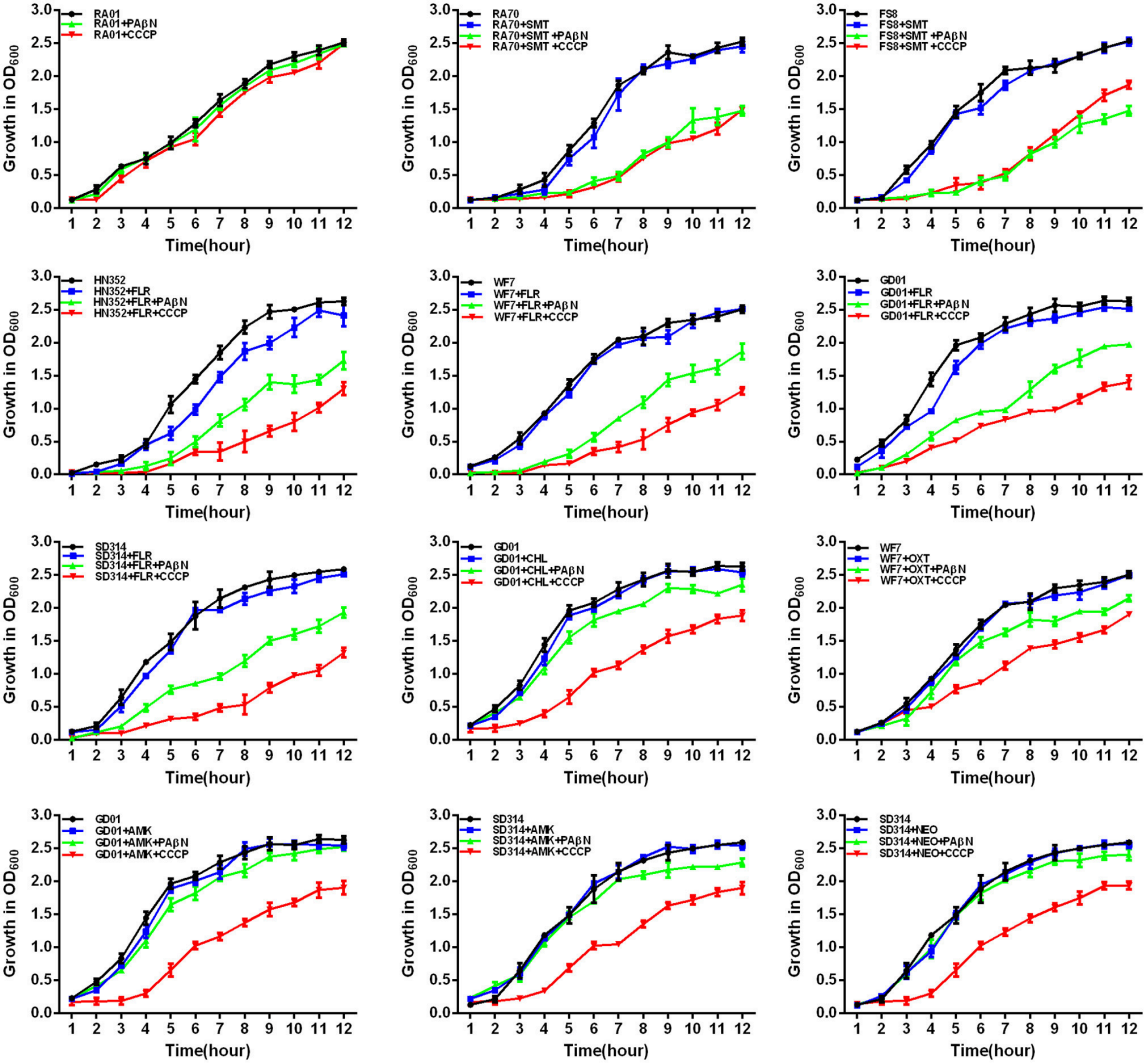
Quantitative drug susceptibility testing of the antibiotics for the *R. anatipestifer* strains, in the presence or absence of each EPI. CCCP (5 μg/mL) and PAβN (40 μg/mL) showed no effects on the growth of *R. anatipestifer* isolates. Strains were grown in the presence of half MIC of each antibiotic as follows: RA70 (32 μg/mL) and FS8 (32 μg/mL) were exposed to SMT, HN352 (8 μg/mL), WF7 (4 μg/mL), GD01 (4 μg/mL), and SD314 (1 μg/mL) were exposed to FLR; GD01 (16 μg/mL) was exposed to CHL; WF7 (4 μg/mL) was exposed to OXT; GD01(16 μg/mL) and SD314 (16 μg/mL) were exposed to AMK; SD314 (32 μg/mL) was exposed to NEO. The figure showed that the *R. anatipestifer* strains grew slowly (between 1 and 12 h) because of the synergistic effect of the antibiotics and EPIs.

**Figure 6:**
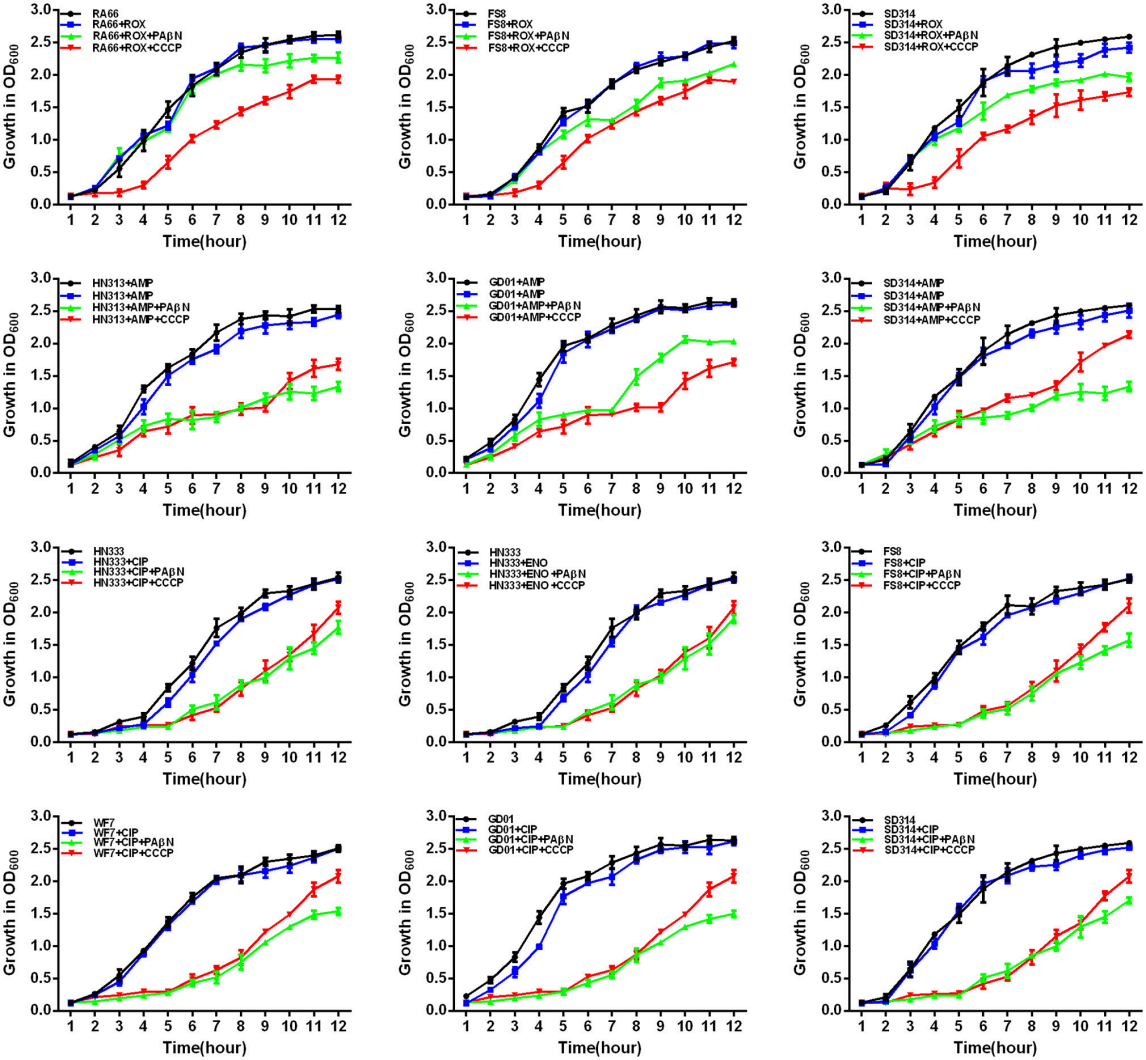
Quantitative drug susceptibility testing of antibiotics for the *R. anatipestifer* strains, in the presence or absence of each EPI. CCCP (5 μg/mL) and PAβN (40 μg/mL) showed no effects on the growth of *R. anatipestifer* isolates. Strains were grown in the presence of half MIC of each antibiotic as follows: RA66 (16 μg/mL), FS8 (8 μg/mL), and SD314 (4 μg/mL) were exposed to ROX; HN313 (8 μg/mL), GD01 (8 μg/mL), and SD314 (4 μg/mL) were exposed to AMP; HN333 (4 μg/mL) was exposed to ENO; HN333 (4 μg/mL), FS8 (2 μg/mL), WF7 (2 μg/mL), GD01 (2 μg/mL), and SD314 (2 μg/mL) were exposed to CIP. The figure showed that the *R. anatipestifer* strains grew slowly (between 1 and 12 h) because of the synergistic effect of the antibiotics and EPIs.

First, the optimal concentrations of PAβN and CCCP at which the growth of bacteria was not influenced were selected. The results showed that the *R. anatipestifer* in the tube containing the antibiotic and EPI grew more slowly than only in the presence of the antibiotic during the evaluation period of 12 h. As evident from the growth curves (Figures 5, 6), with the exception of amphenicols, tetracyclines, and aminoglycosides, all the antimicrobials showed no significant differences in the delay of growth curves between CCCP and PAβN (*P* > 0.05). In comparison with SMT only, the combination of sulfonamides with efflux inhibitors yielded a delay in the growth rates of RA70 and FS8 strains. Similarly, the delay in growth rates in the presence of ciprofloxacin or enrofloxacin indicated a potentiating effect of efflux inhibitors on the activity of the antibiotics. Notably, CCCP and PAβN showed similar effects on the quinolone-resistant isolates. The combination of ROX and an efflux inhibitor induced a delay in the growth of the strains. The effects of CCCP were relatively superior to those of PAβN. The combination of ampicillin and an efflux inhibitor also caused a delay in the growth of the strains; however, PAβN showed superior effects when compared with CCCP.

Notably, aminoglycosides plus PAβN showed no effect on the growth of the strains, whereas when in combination with CCCP, a significant reduction in the growth of the strains was evident (*P* < 0.05). These results showed that the antibiotic activity was definitely enhanced in the presence of an efflux inhibitor, as demonstrated by the delay in the growth of the strains.

## Discussion

Antimicrobials target several essential cellular functions in bacteria, including the biosynthetic pathways of the cell wall, proteins, and nucleic acids, thereby, producing inhibitory and even lethal effects on bacterial survival (Li, X. Z. et al., 2016). In contrast, microorganisms possess remarkable capacities to counteract the action of antimicrobial agents, thereby, conferring resistance. The mechanisms of resistance mainly entail the production of drug inactivating enzymes, alteration of drug targets, mobile resistant genetic elements, and prevention of drug access; this last mechanism refers to the functions of drug efflux and influx (Du Toit, 2017). Efflux is a key mechanism of cellular responses to varied circumstances (Chitsaz and Brown, 2017) and plays a main role in a range of bacterial behaviors, including biofilm formation, quorum sensing, pathogenicity, and virulence. This mechanism allows bacterial cells to regulate their inner environments through the efflux of toxic substances, such as antimicrobial agents and metabolic products.

In China, unfortunately, many domestic ducks frequently share an environment with wild waterfowl, livestock, and humans of the same district. Furthermore, the widespread use of antibiotics to treat related infections has resulted in MDR in *R. anatipestifer*. According to the above theory, it is useful to understand the mechanism by which *R. anatipestifer* develops resistance and how this can improve treatment (Wang Y. et al., 2017).

The development of drug resistance in *R. anatipestifer* has long been correlated with the evolution of resistance genes that code for the drug targets. Several studies have been based on the simple assessment and evaluation of the levels of expression of *R. anatipestifer* EPGs (Li Y. F. et al., 2016; Zhang et al., 2017) and few have determined the effects of efflux inhibitors on the MICs of the antibiotics that are generally used to treat *R. anatipestifer*. Therefore, in the present work, our first aim was to determine whether the combination of antibiotics and an EPI acts synergistically against a set of drug-resistant *R. anatipestifer* strains. The second aim was to reveal the contribution of EP systems to overall drug resistance in these strains.

Based on our clinical investigations, 44 *R. anatipestifer* strains were found to be resistant (data not shown). We selected a set of *R. anatipestifer* strains with different phenotypes and genotypes to study the relationship between the effects of antibiotics and putative EPs that act synergistically. When compared with a few previous reports about the resistance mechanisms of *R. anatipestifer*, the present study focused on the distribution of resistance genes, integrons, and QRDR mutations in *R. anatipestifer* isolates, as shown in Table 3.

Notably, the present study reported that PMQR genes were detected in HN333 (*qnrS*), WF7 (*qnrD*), and SD314 (*qnrS*) isolates. To the best of our knowledge, this is the first report of PMQR resistance genes in *R. anatipestifer*. The chloramphenicol and florfenicol resistance genes *cat* and *floR* detected in this study were also reported in *R. anatipestifer* isolates from Taiwan (Chen et al., 2010, 2012). The aminoglycoside resistance genes, *bla*_*TEM*−1_ (the beta-lactamase resistance gene), *cmlA* (chloramphenicol- and florfenicol-resistance gene), tetracycline and sulfonamide resistance genes were also detected in the *R. anatipestifer* isolates from South China by Sun et al. (2012). Zhu et al. (2018) detected the *tet*(A), *tet*(M), *tet*(Q), *tet*(O), *tet*(B), and *tet*(O/W/32/O) genes in the *R. anatipestifer* isolates from different regions of China (Zhu et al., 2018). In our study, only *tet(*A*)* and *tet*(B) genes were detected. These results show that *R. anatipestifer* has a high diversity of drug resistance genes.

At present, we do not know the exact mechanism by which these compounds inhibit EP activity in *R. anatipestifer*. In order to correlate the data obtained from the MIC determination and efflux activity, we applied a broth microdilution method and growth kinetics techniques to evaluate the synergistic effect between the efflux inhibitors and antibiotics. *R. anatipestifer* has been shown to possess various antibiotic resistance mechanisms. It was hypothesized that the strains that yield higher MICs of the antibiotics would display increased levels of efflux, particularly for their MDR phenotype. However, the clinical isolates also possessed multiple antibiotic resistance genes (see Table 3) that presumably dissimulate the effects of efflux on the phenotype, when was gauged by the MIC. The addition of EPIs highlighted the contribution of EPs to efflux levels in the isolates. In the GD01, SD314, and RA66 isolates, the addition of CCCP, an inhibitor that dissipates the proton motive force required by several EPs, caused a remarkable increase in the accumulation of various antibiotics, such as AMK, NEO, and ROX. This effect led to the slow growth of bacteria and reduced MICs (see Table 1, Figures 5, 6). These findings suggested that reduced efflux and increased antibiotic activity could be attributed to the inhibition of active efflux. The addition of PAβN, an inhibitor of efflux transporters mainly of RND transporters, such as *E. coli* AcrAB-TolC and *P. aeruginosa* MexAB-OprM (Li, X. Z. et al., 2016, chapter 1), also promoted the reduction of the levels of efflux. This was mainly manifested in the synergistic effect of sulfonamide and quinolone antibiotics in the RA70, multidrug-resistant, and extensively drug-resistant strains that caused the slow growth of bacteria and the reduction in MICs (see Table 4, Figures 5, 6), since PAβN is not a specific inhibitor of RND EPs. However, this reduction was not as great as that seen with CCCP, suggesting that either the RND pumps associated with efflux in *R. anatipestifer* were limited or CCCP mediated other cellular activities in *R. anatipestifer*. Furthermore, the efflux inhibitors possibly acted as helper compounds of antibiotic activity in *R. anatipestifer* treatment. Thus, the antibiotics could concentrate and penetrate the bacteria, thereby, increasing their effective concentrations. The combined use of antibiotics and EPIs in the control and prevention of *R. anatipestifer* therapy would be beneficial for the development of new therapeutic strategies for *R. anatipestifer* infection.

For the analysis of putative EP gene expression, we first compared the mRNA levels of the *R. anatipestifer* clinical strains with the antibiotic susceptible reference strain (RA01) following exposure to the antibiotics and EPIs. All strains presented an intricate resistance pattern to the antibiotics tested (Tables 1, 2), due to the presence of the resistance gene (Table 3), coupled with a component of efflux, as demonstrated by the reduction in resistance levels with the EPIs (Table 4). No EP gene was overexpressed in the RA01 strain (used as a reference; Figure 1). The EPGs, *RIA-1853* and *RIA-0286*, were overexpressed in the presence of AMP and AMK, demonstrating the contribution of these EPs to the resistance phenotype of the HN313, GD01, and SD314 strains (Figures 1, 3, 4). The addition of CCCP to AMK caused a significant reduction in the expression of *RIA-1853* and *RIA-0286*. Furthermore, the addition of PAβN to AMP also significantly reduced the expression of *RIA-1853* and *RIA-0286*.

The EPGs, *RIA-1993, RIA-1069*, and *RIA-1614*, were overexpressed in the presence of OXT in the WF7 strain. Following the addition of CCCP or PAβN to OXT, a significant reduction was observed in the expression of those genes. These EPGs might be associated with aminoglycoside, β-lactam, and tetracycline resistance in particular strains. We noticed a general overexpression of almost all the EPGs in the resistant strains upon exposure to quinolones. These results indicated that the activity of *R. anatipestifer* EPs was multifunctional and was associated with the outflow of drugs in response to a particular gene.

The RT-qPCR data combined with the real time growth kinetics and the results of MICs showed that the response of the resistant clinical strains were efflux-mediated. Furthermore, we found a linear correlation between EP gene expression and the reduction in antibiotic resistance induced by the EPIs, such as that observed on exposure to aminoglycosides. In addition, we noticed that some genes were upregulated upon exposure to the combination of antibiotics and EPIs (see Figure 1, for example HN333-QUO), this might reflect another mechanism of action of the EPIs in *R. anatipestifer*. These findings also suggested that the EPs of clinical strains of *R. anatipestifer* had diverse substrate specificities. The results suggested that the RND and MFS EPs might play a major role in *R. anatipestifer* isolates. To explore the details of the mechanisms of EP resistance and to test these hypotheses, further experiments would be necessary.

In summary, to our knowledge, this is the first report to investigate the expression levels of EPGs. The rationale and procedures employed have proved to be useful in evaluating the widespread existence of active efflux systems in drug-resistant *R. anatipestifer* strains. This study supported the concept that intrinsic efflux activity also contributes to overall resistance in drug-resistant clinical strains of *R. anatipestifer*. Our results also showed that EPIs, such as PAβN and CCCP, inhibited efflux activity to enhance the clinical effects of antibiotics. It is noteworthy that during the process of clinical treatment, antibiotics were mixed with various antibacterial effects, or the effects of antibiotics and EPIs were superimposed on each other. This study has demonstrated the utility of EPIs in the screening for novel therapeutic options to combat drug resistance in *R. anatipestifer* and has shown that the combination of antibiotics and EPIs should be further investigated for effective clinical applications.

## Author contributions

QC and FZ conceived and designed the study. QC, GJ, SL, and XG performed the experiments. QC, XG, FZ, LS, SS, and YL analyzed the date. QC wrote the manuscript. All authors reviewed and approved the final version of the manuscript.

### Conflict of interest statement

The authors declare that the research was conducted in the absence of any commercial or financial relationships that could be construed as a potential conflict of interest.

## Supplementary material

Figure S1 Assessment of the effect of EPIs on the accumulation of EB on the *Riemerella anatipestifer* strains used in the study. Fluorometric EB efflux assay. Ethidium bromide was used at the equilibrium concentration for each strain as follows: 0.3 μg/mL for RA01; 0.5 μg/mL for RA70, HN352, RA66, and HN313 strains. Each EPI was tested at half MIC; CCCP (5 μg/mL) and PAβN (40 μg/mL) showed no effects on the growth of *R. anatipestifer* isolates. Dotted line means strains assayed with 5% calf serum + PBS; red line means strains assayed with PBS; green line means strains assayed with 5% calf serum + PBS + PAβN; yellow line means strains assayed with 5% calf serum + PBS + CCCP.

Figure S2 Assessment of the effect of EPIs on the accumulation of EB on the *Riemerella anatipestifer* strains used in the study. Fluorometric EB efflux assay. Ethidium bromide was used at the equilibrium concentration for each strain as follows: 0.6 μg/mL for the HN333 strain; and 1.0 μg/mL for FS8, WF7, GD01, and SD314 strains. Each EPI was tested at half MIC; CCCP (5 μg/mL) and PAβN (40 μg/mL) showed no effects on the growth of *R. anatipestifer* isolates. Dotted line means strains assayed with 5% calf serum + PBS; red line means strains assayed with PBS; green line means strains assayed with 5% calf serum + PBS + PAβN; yellow line means strains assayed with 5% calf serum + PBS + CCCP.

Table S1 Minimal iinhibitory concentration interpretive standards for *R. anatipestifer* strains studied.

Table S2 PCR primers used in this study.

Table S3 Table S3 RT-qPCR primers used in this study.

Table S4 Putative efflux transporters described in *Riemerella anatipestifer* and their substrates.
