# Supplementary material for: Interplay Between the Phenotype and Genotype, and Efflux Pumps in Drug-Resistant Strains of Riemerella anatipestifer
Source: Front Microbiol. 2018 Oct 1;9:2136. doi: 10.3389/fmicb.2018.02136 (PMC6174861; doi:10.3389/fmicb.2018.02136)
Supplement: Table S1 — Minimal iinhibitory concentration interpretive standards for R. anatipestifer strains studied. [file Table_1.DOCX]

Supplemental material Table 1 Minimal Inhibitory Concentration Interpretive Standards for *R.anatipestifer* strains studied

| Antibiotics | MIC Interpretive Criteria  (µg/mL) | | |
| --- | --- | --- | --- |
|  | Susceptible | Intermediate | Resistant |
| Streptomycin | ≤4 | 8 | ≥16 |
| Kanamycin | ≤8 | 16 | ≥32 |
| Gentamicin | ≤4 | 8 | ≥16 |
| Spectinomycin | ≤4 | 8 | ≥16 |
| Amikacin | ≤4 | 8 | ≥16 |
| Neomycin | ≤8 | 16 | ≥32 |
| Tobramycin | ≤4 | 8 | ≥16 |
| Nalidixic acid | ≤4 | 8 | ≥16 |
| Ciprofloxacin | ≤1 | 2 | ≥4 |
| Enrofloxacin | ≤0.5 | 1 | ≥2 |
| Ampicillin | ≤2 | 3 | ≥4 |
| Cefoxitin | ≤0.5 | 0.75 | ≥1 |
| Chloramphenicol | ≤4 | 6 | ≥8 |
| Florfenicol | ≤1 | 2 | ≥4 |
| Sulfamonomethoxine | ≤8 | 16 | ≥32 |
| Roxithromycin | ≤2 | 4 | ≥8 |
| Tetracycline | ≤1 | 2 | ≥4 |
| Oxytetracycline | ≤1 | 2 | ≥4 |
