# Supplementary material for: Interplay Between the Phenotype and Genotype, and Efflux Pumps in Drug-Resistant Strains of Riemerella anatipestifer
Source: Front Microbiol. 2018 Oct 1;9:2136. doi: 10.3389/fmicb.2018.02136 (PMC6174861; doi:10.3389/fmicb.2018.02136)
Supplement: Table S2 — PCR primers used in this study. [file Table_2.DOC]

Supplemental material Table 2 PCR primers used in this study.

| Resistance gene | Primer | Sequence (5′-3′) | Amplicon size (bp) | Annealing  Temperature (°C ) | Primer reference |
| --- | --- | --- | --- | --- | --- |
| *ermA* | ermA-F | CTTCGATAGTTTATTAATATTAGT | 645 | 50 | 1 |
|  | ermA-R | TCTAAAAAGCATGTAAAAGAA |  |  |  |
| *ermB* | ermB-F | AGTAACGGTACTTAAATTGTTTAC | 639 | 57 | 1 |
|  | ermB-R | GAAAAGGATCTCAACCAAATA |  |  |  |
| *ermC* | ermC-F | GCTAATATTGTTTAAATCGTCAAT | 642 | 50 | 1 |
|  | ermC-R | TCAAAACATAATATAGATAAA |  |  |  |
| *ermF* | ermF-F | ACCACTTTCCAGTCTTACGAAG | 994 | 55 | 2,3 |
|  | ermF-R | CGACTTTGAACTACGAAGGATG |  |  |  |
| *bla_CMY-2_* | bla_CMY-2_-F | ATGATGAAAAAATCGTTATGC | 1143 | 55 | 4 |
|  | bla_CMY-2_-R | TTGCAGCTTTTCAAGAATGCG |  |  |  |
| *bla*_TEM_ | bla_TEM_-F | ATAAAATTCTTGAAGACGAAA | 1080 | 52 | 5 |
|  | bla_TEM_-R | GACAGTTACCAATGCTTAATC |  |  |  |
| *bla*_SHV_ | bla_SHV_-F | CACTCAAGGATGTATTGTG | 885 | 55 | 5 |
|  | bla_SHV_-R | TTAGCGTTGCCAGTGCTCG |  |  |  |
| *bla*_DHA_ | bla_DHA_-F | AACTTTCACAGGTGTGCTGT | 387 | 56 | 5 |
|  | bla_DHA_-R | CCGTACGCATACTGGCTTTC |  |  |  |
| *bla*_CTX-M-1G_ | bla_CTX-M-1G_-F | CTTCCAGAATAAGGAATCCC | 949 | 55 | 5 |
|  | bla_CTX-M-1G_-R | CGTCTAAGGCGATAAACAAA |  |  |  |
| *bla*_CTX-M-9G_ | bla_CTX-M-9G_-F | TGACCGTATTGGGAGTTTG | 902 | 58.5 | 5 |
|  | bla_CTX-M-9G_-R | ACCAGTTACAGCCCTTCG |  |  |  |
| *bla*_NDM-1_ | *bla*_NDM-1_-F | GGCCAGCAAATGGAAACTGG | 443 | 55 | This study |
|  | *bla*_NDM-1_-R | AATACCTTGAGCGGGCCAAA |  |  |  |
| *aac(3’)-Ⅰa* | aac(3')-Ⅰa-F | TTACGCAGCAGCAACGATGT | 402 | 58.5 | 6 |
|  | aac(3')-Ⅰa-R | GTTGGCCTCATGCTTGAGGA |  |  |  |
| *aac(3’)-Ⅱc* | aac(3')-Ⅱc-F | AACCGGTGACCTATTGATGG | 774 | 58.5 | 7 |
|  | aac(3')-Ⅱc-R | TGTGCTGGCACGATCGGAGT |  |  |  |
| *aac(3’)-Ⅳ* | aac(3')-Ⅳ-F | GGCCACTTGGACTGATCGAG | 609 | 58.5 | 7 |
|  | aac(3')-Ⅳ-R | GCGGATGCAGGAAGATCAAC |  |  |  |
| *aph(2')-Ⅰb* | aph(2')-Ⅰb- F | TGGTTAACTTGGACGCTGAG | 720 | 56.5 | 7 |
|  | aph(2')-Ⅰb-R | TCCTGCCAAATTCTTTCCCG |  |  |  |
| *aph(3')-Ⅱ* | aph(3')-Ⅱ-F | TCTGAAACATGGCAAAGGTAG | 582 | 54 | 7 |
|  | aph(3')-Ⅱ-R | AGCCGTTTCTGTAATGAAGGA |  |  |  |
| *aph(3')-Ⅳ* | aph(3')-Ⅳ-F | AGAACGAGATGACGTTGGAG | 1037 | 56.5 | 7 |
|  | aph(3')-Ⅳ-R | AGTTGGTCAAGACCAATGCG |  |  |  |
| *aph(3')-Ⅶ* | aph(3')-Ⅶ-F | TCCATAGGATGGCAAGATCC | 690 | 56.5 | 7 |
|  | aph(3')-Ⅶ-R | TTCAACGGGAAACGTCTTGC |  |  |  |
| *aph(4’)-Ⅰa* | aph(4')-Ⅰa-F | TCCGGAAGTGCTTGACATTG | 540 | 58.5 | 7 |
|  | aph(4')-Ⅰa-R | GGATGCCTCCGCTCGAAGTA |  |  |  |
| *aadA1* | aadA1-F | AGGTAGTTGGCGTCATCGAG | 589 | 58.5 | 7 |
|  | aadA1-R | CAGTCGGCAGCGACATCCTT |  |  |  |
| *aadA2* | aadA2-F | GGTGCTAAGCGTCATTGAGC | 470 | 51 | 6 |
|  | aadA2-R | GCTTCAAGGTTTCCCTCAGC |  |  |  |
| *aadB* | aadB-F | GCGAAATCTGCCGCTCTG | 412 | 58 | 5 |
|  | aadB-R | TGCGAGCCTGTAGGACTC |  |  |  |
| *rmtA* | rmtA-F | CTAGCGTCCATCCTTTCCTC | 635 | 55 | 8 |
|  | rmtA-R | TTGCTTCCATGCCCTTGCC |  |  |  |
| *rmtB* | rmtB-F | ACATCAACGATGCCCTCAC | 724 | 54 | 9 |
|  | rmtB-R | AAGTTCTGTTCCGATGGTC |  |  |  |
| *rmtC* | rmtC-F | CGAAGAAGTAACAGCCAAAG | 711 | 55 | 8 |
|  | rmtC-R | ATCCCAACATCTCTCCCACT |  |  |  |
| *rmtD* | rmtD-F | CGGCACGCGATTGGGAAGC | 401 | 55 | 8 |
|  | rmtD-R | CGGAAACGATGCGACGAT |  |  |  |
| *rmtE* | rmtE-F | ATGAATATTGATGAAATGGTTGC | 1056 | 55 | 8,10 |
|  | rmtE-R | TGATTGATTTCCTCCGTTTTTG |  |  |  |
| *armA* | armA-F | CAATCAGGGGCAGTTATCA | 528 | 54 | 9 |
|  | armA-R | CCCTATAACCTTCGAATC |  |  |  |
| *npmA* | npmA-F | AGGGCTATCTAATGTGGTG | 207 | 52 | 7 |
|  | npmA-R | TATTTCCGCTTCTTCGTAT |  |  |  |
| *cat1* | cat1-F | CTTGTCGCCTTGCGTATAAT | 508 | 54 | 11 |
|  | cat1-R | ATCCCAATGGCATCGTAAAG |  |  |  |
| *cat2* | cat2-F | AACGGCAYGATGAACCTGAA | 547 | 50 | 11 |
|  | cat2-R | ATCCCAATGGCATCGTAAAG |  |  |  |
| *cmlA* | cmlA-F | CGCCACGGTGTTGTTGTTAT | 394 | 55 | 11 |
|  | cmlA-R | GCGACCTGCGTAAATGTCAC |  |  |  |
| *cmlB* | cmlB-F | ACTCGGCATGGACATGTACT | 840 | 55 | 11 |
|  | cmlB-R | ACGGACTGCGGA ATCCATAG |  |  |  |
| *floR* | floR-F | CTGAGGGTGTCGTCATCTAC | 673 | 58 | 12 |
|  | floR-R | GCTCCGACAATGCTGACTAT |  |  |  |
| *tet*( A) | tetA-F | GCGCCTTTCCTTTGGGTTCT | 831 | 51 | 11 |
|  | tetA-R | CCACCCGTTCCACGTTGTTA |  |  |  |
| *tet*( B) | tetB-F | CATTAATAGGCGCATCGCTG | 930 | 55 | 13 |
|  | tetB-R | TGAAGGTCATCGATAGCAGG |  |  |  |
| *tet*( C) | tetC-F | GCTGTAGGCATAGGCTTGGT | 888 | 55 | 13 |
|  | tetC-R | GCCGGAAGCGAGAAGAATCA |  |  |  |
| *tet* ( K) | tetK-F | TTA GGT GAA GGG TTA GGT CC | 718 | 55 | 14 |
|  | tetK-R | GCA AAC TCA TTC CAG AAG CA |  |  |  |
| *tet*( M) | tetM-F | GTT AAA TAG TGT TCT TGG AG | 647 | 55 | 14 |
|  | tetM-R | CTA AGA TAT GGC TCT AAC AA |  |  |  |
| *sul1* | sul1-F | GTGACGGTGTTCGGCATTCT | 779 | 68 | 13 |
|  | sul1-R | CCGAGAAGGTGATTGCGCT |  |  |  |
| *sul2* | sul2-F | CCTGTTTCGTCCGACACAGA | 435 | 51 | 11 |
|  | sul2-R | GAAGCGCAGCCGCAATTCAT |  |  |  |
| *sul3* | sul3-F | GAGCAAGATTTTTGGAATCG | 880 | 55 | 13 |
|  | sul3-R | CATCTGCAGCTAACCTAGGGCTTTGGA |  |  |  |
| *gyrA* | gyrA-F | AGAGAAGGGTTTTGTATGG | 241 | 55 | 7 |
|  | gyrA-R | GGGGGCTTCAGTATAACGCA |  |  |  |
| *gyrB* | gyrB-F | AAATAGGTGCTGGTGGTA | 1486 | 54 | 12 |
|  | gyrB-R | CAAAGTTGTTCTGGGTTC |  |  |  |
| *parC* | parC-F | TCTATACTGGCGACTCCG | 1707 | 55 | 5 |
|  | parC-R | CAGCATTAAGCCTTCTCA |  |  |  |
| *parE* | parE-F | GTGGAACGCACCTCAATG | 782 | 56 | 7 |
|  | parE-R | GCTCCTTACCCTAAACAACG |  |  |  |
| *qnrA* | qnrA-F | ATTTCTCACGCCAGGATTTG | 516 | 55 | 15 |
|  | qnrA-R | GATCGGCAAAGGTTAGGTCA |  |  |  |
| *qnrB* | qnrB-F | GATCGTGAAAGCCAGAAAGG | 469 | 55 | 15 |
|  | qnrB-R | ACGATGCCTGGTAGTTGTCC |  |  |  |
| *qnrC* | qnrC-F | ATTTCTCACAGGCAAACT | 666 | 54 | 7 |
|  | qnrC-R | CTGGAATAACAATCACCC |  |  |  |
| *qnrD* | qnrD-F | TTTTCGCTAACTAACTCGC | 984 | 54 | 13 |
|  | qnrD-R | GAAAGGATAAACAGGCAAAT |  |  |  |
| *qnrS* | qnrS-F | ACGACATTCGTCAACTGCAA | 417 | 55 | 15 |
|  | qnrS-R | TAAATTGGCACCCTGTAGGC |  |  |  |
| *qepA* | qepA-F | GCAGGTCCAGCAGCGGGTAG | 199 | 60 | 16 |
|  | qepA-R | CTTCCTGCCCGAGTATCGTG |  |  |  |
| *oqxA* | oqxA-F | GATCAGTCAG TGGGATAGTTT | 670 | 52 | 17 |
|  | oqxA-R | TACTCGGCGTTAACTGATTA |  |  |  |
| *oqxB* | oqxB-F | TTCTCCCCCGGCGGGAAGTAC | 512 | 52 | 14 |
|  | oqxB-R | CTCGGCCATTTTGGCGCGTA |  |  |  |
| *aac(6’)-Ib* | aac(6')-Ib-F | TTGCGATGCTCTATGAGTGGCTA | 482 | 54 | 6 |
|  | aac(6')-Ib-R | CTCGAATGCCTGGCGTGTTT |  |  |  |
| *mcr-1* | Mcr-1-F | GCGAGTGTTGCCGTTTTCTT | 1148 | 53 | This study |
|  | Mcr-1-R | CGCTTAAAATACGCAGGCCC |  |  |  |
| Variable region class 1 | Var-1F | GGCATCCAAGCAGCA AG | variabe | 58 | 18 |
|  | Var-1R | AAGCAGACTTGACCTGA |  |  |  |
| *IntI1* | intI1-F | CCTCCCGCACGATGATC | 280 | 55 | 18 |
|  | intI1-R | TCCACGCATCGTCAGGC |  |  |  |
| Variable region class 2 | Var-2F | CGGGATCCCGGACGGCATGCACGATTTGTA | variabe | 58.5 | 18 |
|  | Var-2R | GATGCCATCGCAAGTACGAG |  |  |  |
| *IntI2* | intI2-F | TTATTGCTGGGATTAGGC | 233 | 50 | 18,19 |
|  | intI2-R | ACGGCTACCCTCTGTTATC |  |  |  |
| Variable region class 3 | Var-3F | CGGGATCCCGGACGGCATGCACGATTTGTA | variabe | 60 | 19,20 |
|  | Var-3R | GATGCCATCGCAAGTACGAG |  |  |  |
| *IntI3* | intI3-F | AGTGGGTGGCGAATGAGTG | 600 | 50 | 19 |
|  | intI3-R | TGTTCTTGTATCGGCAGGTG |  |  |  |

**References**

1. Shigemura K, Osawa K, Miura M, et al. Azithromycin resistance and its mechanism in Neisseria gonorrhoeae strains in Hyogo, Japan. Antimicrob Agents Chemother 2015;59:2695-2699.

2. Liu M, Zhang L, Huang L, et al. Use of Natural Transformation To Establish an Easy Knockout Method in Riemerella anatipestifer. Appl Environ Microbiol 2017;83.

3. Xing L, Yu H, Qi J, et al. ErmF and ereD are responsible for erythromycin resistance in Riemerella anatipestifer. PLoS One 2015;10:e0131078.

4. Yan JJ, Hong CY, Ko WC, et al. Dissemination of blaCMY-2 among Escherichia coli isolates from food animals, retail ground meats, and humans in southern Taiwan. Antimicrob Agents Chemother 2004;48:1353-1356.

5. Liu JH, Wei SY, Ma JY, et al. Detection and characterisation of CTX-M and CMY-2 beta-lactamases among Escherichia coli isolates from farm animals in Guangdong Province of China. Int J Antimicrob Agents 2007;29:576-581.

6. Yang FF, Sun YN, Li JX, et al. Detection of aminoglycoside resistance genes in Riemerella anatipestifer isolated from ducks. Vet Microbiol 2012;158:451-452.

7. Sun N, Liu JH, Yang F, et al. Molecular characterization of the antimicrobial resistance of Riemerella anatipestifer isolated from ducks. Vet Microbiol 2012;158:376-383.

8. Doi Y, Arakawa Y. 16S ribosomal RNA methylation: emerging resistance mechanism against aminoglycosides. Clin Infect Dis 2007;45:88-94.

9. Chen L, Chen ZL, Liu JH, et al. Emergence of RmtB methylase-producing Escherichia coli and Enterobacter cloacae isolates from pigs in China. J Antimicrob Chemother 2007;59:880-885.

10. Davis MA, Baker KN, Orfe LH, et al. Discovery of a gene conferring multiple-aminoglycoside resistance in Escherichia coli. Antimicrob Agents Chemother 2010;54:2666-2669.

11. Chen S, Zhao S, White DG, et al. Characterization of multiple-antimicrobial-resistant salmonella serovars isolated from retail meats. Appl Environ Microbiol 2004;70:1-7.

12. Chen YP, Lee SH, Chou CH, et al. Detection of florfenicol resistance genes in Riemerella anatipestifer isolated from ducks and geese. Vet Microbiol 2012;154:325-331.

13. Boerlin P, Travis R, Gyles CL, et al. Antimicrobial resistance and virulence genes of Escherichia coli isolates from swine in Ontario. Appl Environ Microbiol 2005;71:6753-6761.

14. Kim TJ, Na YR, Lee JI. Investigations into the basis of chloramphenicol and tetracycline resistance in Staphylococcus intermedius isolates from cases of pyoderma in dogs. J Vet Med B Infect Dis Vet Public Health 2005;52:119-124.

15. Robicsek A, Strahilevitz J, Sahm DF, et al. qnr prevalence in ceftazidime-resistant Enterobacteriaceae isolates from the United States. Antimicrob Agents Chemother 2006;50:2872-2874.

16. Yamane K, Wachino J, Suzuki S, et al. Plasmid-mediated qepA gene among Escherichia coli clinical isolates from Japan. Antimicrob Agents Chemother 2008;52:1564-1566.

17. Hansen LH, Sorensen SJ, Jorgensen HS, et al. The prevalence of the OqxAB multidrug efflux pump amongst olaquindox-resistant Escherichia coli in pigs. Microb Drug Resist 2005;11:378-382.

18. Zheng F, Lin G, Zhou J, et al. Discovery and characterization of gene cassettes-containing integrons in clinical strains of Riemerella anatipestifer. Vet Microbiol 2012;156:434-438.

19. Goldstein C, Lee MD, Sanchez S, et al. Incidence of class 1 and 2 integrases in clinical and commensal bacteria from livestock, companion animals, and exotics. Antimicrob Agents Chemother 2001;45:723-726.

20. Diaz-Mejia JJ, Amabile-Cuevas CF, Rosas I, et al. An analysis of the evolutionary relationships of integron integrases, with emphasis on the prevalence of class 1 integrons in Escherichia coli isolates from clinical and environmental origins. Microbiology 2008;154:94-102.
