# Supplementary material for: Interplay Between the Phenotype and Genotype, and Efflux Pumps in Drug-Resistant Strains of Riemerella anatipestifer
Source: Front Microbiol. 2018 Oct 1;9:2136. doi: 10.3389/fmicb.2018.02136 (PMC6174861; doi:10.3389/fmicb.2018.02136)
Supplement: Table S3 — RT-qPCR primers used in this study. [file Table_3.DOC]

Supplemental material Table 3 RT-qPCR primers used in this study.

| Putative efflux gene | Primer | Sequence (5′-3′) | Amplicon size (bp) | Primer reference |
| --- | --- | --- | --- | --- |
| RIA-1800 | 1F | TGCCAATCAGTACAGTGCCT | 150 | This study |
|  | 1R | ATACAGAAGCGGTTTGGTGGT |  |  |
| RIA-1853 | 2F | CGTCATAAGAGGCGGACCAA | 137 | This study |
|  | 2R | GTTTCGGAGGGATTGCAGGA |  |  |
| RIA-0245 | 3F | TAGCCACGCTTCTAGGTTGG | 113 | This study |
|  | 3R | GCCTGCGTATAGGGCAGTAG |  |  |
| RIA-0257 | 4F | TTCTCGTCTGTTGTCGCCTC | 160 | This study |
|  | 4R | GTATGGGAGCTTGGGTGCTT |  |  |
| RIA-0437 | 5F | ACGGAACTGCACTCACAAGG | 152 | This study |
|  | 5R | TACCGCTTGGTATTGCTCGT |  |  |
| RIA-0577 | 6F | CCGCTAACCACCCACCAATA | 178 | This study |
|  | 6R | TTCACCGAGATGTGGGAACG |  |  |
| RIA-0746 | 7F | GACCTTAGCCTTCGGGTTGG | 175 | This study |
|  | 7R | AGTTTCGGGGCTTCCCATTT |  |  |
| RIA-1554 | 8F | GAGCGTGGTGGTTTGGTTTT | 148 | This study |
|  | 8R | ACCACCCAGAAACCACCTTG |  |  |
| RIA-1117 | 9F | CCACCTCCCAATCTGCTTGTA | 103 | This study |
|  | 9R | AGTGAAGGTGCAGGAGATGC |  |  |
| RIA-1118 | 10F | CCCATTGCTTCTTGCGTAGC | 113 | This study |
|  | 10R | GTGATGGCGATAGGTGTGGT |  |  |
| RIA-1215 | 11F | AAGCCTAGCGTGGTAGATGC | 149 | This study |
|  | 11R | TCAAATGTTGCCGTCCCGTA |  |  |
| RIA-1993 | 12F | AAACTGGCAACATCGGCAAC | 104 | This study |
|  | 12R | GGCTACGAGCACCGTTATGA |  |  |
| RIA-0286 | 13F | TTCAGGTTACAGCGTTGGGG | 198 | This study |
|  | 13R | ATCGTCTCACCAGAAGCGTG |  |  |
| RIA-1069 | 14F | CAACGGCTTCCCTCCTGATT | 180 | This study |
|  | 14R | TTGATAACGGGGTGATGCGT |  |  |
| RIA-1614 | 15F | GCGACTACAGCTCCCACAAA | 123 | This study |
|  | 15R | AATAAAACGCCCGCAGAAGC |  |  |

The protocol used comprised the following amplification program: reverse transcription for 15 min at 50°C, followed by 2 min at 85°C, an initial denaturation step for 5 min at 95°C, 40 cycles of extension at 95°C for 10 s, and 60°C for 30 s.
