# Supplementary material for: Interplay Between the Phenotype and Genotype, and Efflux Pumps in Drug-Resistant Strains of Riemerella anatipestifer
Source: Front Microbiol. 2018 Oct 1;9:2136. doi: 10.3389/fmicb.2018.02136 (PMC6174861; doi:10.3389/fmicb.2018.02136)
Supplement: Table S4 — Putative efflux transporters described in Riemerella anatipestifer and their substrates. [file Table_4.DOC]

Supplemental material Table 4 Putative efflux transporters described in *Riemerella anatipestifer* and their substrates.

| Gene | Function | Substrates | Family | Energy source | Reference |
| --- | --- | --- | --- | --- | --- |
| RIA1800 | Probable membrane transporter of cations and cationic drugs; Probable QacE family quaternary ammonium compound efflux | SMT, CIP | SMR | PMF | 1 |
| RIA0245 | Probable major facilitator family transporter | ROX | MFS | PMF | 1 |
| RIA0257 | Probable MFS transporter | FLR, CCCP | MFS | PMF | 1 |
| RIA0437 | putative proton dependent di-tripeptide transporter | AMK | MFS | PMF | 1 |
| RIA0577 | Probable Dipeptide/tripeptide permease | FLR | MFS | PMF | 1 |
| RIA0746 | Probable MFS transporter or glucose-galactose transporter | CIP, ROX | MFS | PMF | 1 |
| RIA1554 | Probable major facilitator family transporter | AMP | MFS | PMF | 1 |
| RIA1853 | Probable tetracycline resistance MFS efflux pump | FLR, AMP, CIP, PAβN | MFS | PMF | 1 |
| RIA1117 | Probable RND efflux system outer membrane lipoprotein | CIP, CCCP, PAβN | RND | PMF | In this study |
| RIA1118 | Probable hydrophobe/amphiphile efflux-1 family RND transporter | FLR, SMT | RND | PMF | In this study |
| RIA1215 | Probable efflux RND transporter periplasmic adaptor subunit | AMK | RND | PMF | In this study |
| RIA1993 | Putative silver efflux pump or CusA/CzcA family heavy metal efflux RND transporter | PAβN, CCCP, SMT, FLR, OXT | RND | PMF | In this study |
| RIA0286 | Possible MATE family efflux transporter | AMP, CCCP | MATE | PMF | In this study |
| RIA1069 | Probable Outer membrane efflux protein | CCCP, PAβN | unkown | - | In this study |
| RIA1614 | Probable ABC-type transport system involved in resistance to organic solvents, permease component | CHL, OXT, ROX | ABC | ATP | In this study |

Based on the R. anatipestifer RA-GD genome (accession no. CP002562.1), gene is Locus tag.

ABC, ATP-binding cassette; AMK, amikacin; AMP, ampicillin; AP, antimicrobial peptides; ATP, adenosine triphosphate; CIP, Ciprofloxacin; CHL, chloramphenicol; DOX, Doxycycline; EtBr, ethidium bromide; ENO, Enrofloxacin; FLR, florfenicol；GEN, gentamicin; KAN, kanamycin; MFS, major facilitator superfamily; MATE, multidrug and toxic compound extrusion Family; NA, nalidixic acid; NEO, neomycin; OXT, Oxytetracycline; PMF, proton motive force; RND, resistance nodulation division; SMT, Sulfamonomethoxine; ROX, Roxithromycin; SMR, smallmultidrug resistance; SPE, spectinomycin; STR, streptomycin; TET, tetracycline; TOB, Tobramycin

Reference

1. Wang X, Zhu D, Wang M, et al. Complete genome sequence of Riemerella anatipestifer reference strain. J Bacteriol 2012;194:3270-3271.
